# The Impact of *N*^ε^-Acryloyllysine Piperazides on the Conformational Dynamics of Transglutaminase 2

**DOI:** 10.3390/ijms24021650

**Published:** 2023-01-13

**Authors:** Andreas Heerwig, Alfred Kick, Paul Sommerfeld, Sophia Eimermacher, Frederick Hartung, Markus Laube, Dietmar Fischer, Hans-Jürgen Pietzsch, Jens Pietzsch, Reik Löser, Michael Mertig, Markus Pietsch, Robert Wodtke

**Affiliations:** 1 Kurt-Schwabe-Institut für Mess- und Sensortechnik Meinsberg e.V., 04736 Waldheim, Germany; 2 Institutes I & II of Pharmacology, Center of Pharmacology, Faculty of Medicine and University Hospital of Cologne, University of Cologne, 50931 Cologne, Germany; 3 Helmholtz-Zentrum Dresden-Rossendorf, Institute of Radiopharmaceutical Cancer Research, Bautzner Landstraße 400, 01328 Dresden, Germany; 4 School of Science, Faculty of Chemistry and Food Chemistry, Technische Universität Dresden, 01062 Dresden, Germany

**Keywords:** transamidase inhibitor, DNA lever, binding kinetics, conformational change

## Abstract

In addition to the classic functions of proteins, such as acting as a biocatalyst or binding partner, the conformational states of proteins and their remodeling upon stimulation need to be considered. A prominent example of a protein that undergoes comprehensive conformational remodeling is transglutaminase 2 (TGase 2), the distinct conformational states of which are closely related to particular functions. Its involvement in various pathophysiological processes, including fibrosis and cancer, motivates the development of theranostic agents, particularly based on inhibitors that are directed toward the transamidase activity. In this context, the ability of such inhibitors to control the conformational dynamics of TGase 2 emerges as an important parameter, and methods to assess this property are in great demand. Herein, we describe the application of the switchSENSE^®^ principle to detect conformational changes caused by three irreversibly binding *N*^ε^-acryloyllysine piperazides, which are suitable radiotracer candidates of TGase 2. The switchSENSE^®^ technique is based on DNA levers actuated by alternating electric fields. These levers are immobilized on gold electrodes with one end, and at the other end of the lever, the TGase 2 is covalently bound. A novel computational method is introduced for describing the resulting lever motion to quantify the extent of stimulated conformational TGase 2 changes. Moreover, as a complementary biophysical method, native polyacrylamide gel electrophoresis was performed under similar conditions to validate the results. Both methods prove the occurrence of an irreversible shift in the conformational equilibrium of TGase 2, caused by the binding of the three studied *N*^ε^-acryloyllysine piperazides.

## 1. Introduction

The original assumption of proteins as static objects has considerably changed with the investigations of oxygen and carbon monoxide binding to myoglobin and hemoglobin. It culminated in the current perception that proteins are conformationally dynamic molecules and pass through an ensemble of conformations even in their native states [1,2]. Accordingly, a thermodynamic concept was formulated using the conformational free energy landscape, describing conformational sub-states and proteins’ fluctuations [2,3,4]. In light of biochemical processes, remodeling of the conformational ensemble of proteins is a central aspect of cellular signaling [5]. It could also be considered a discrete signaling principle [6].

Conformational flexibility is an inherent characteristic of proteins. However, the kind and extent of motions can differ, ranging from bond vibrations and side-chain rotations to the collective movement of domains [3,7]. Regarding the latter protein motion, a protein known to undergo significant conformational remodeling is transglutaminase 2 (TGase 2). TGase 2 is an enzyme that catalyzes the Ca^2+^-dependent post-translational modification of proteins via acyl transfer of protein-bound glutamine residues to primary amines (transamidase activity), including protein-bound lysine residues and low-molecular-weight biogenic amines and polyamines [8,9]. In addition to this enzymatic function, further enzymatic and non-enzymatic functions have been attributed to TGase 2. For example, TGase 2 binds and hydrolyzes GTP and acts as the Gα_h_ subunit of the dimeric G_h_ protein [10,11,12]. In this context, Ca^2+^ and GDP/GTP serve as allosteric regulators of the GTP-binding function and the transamidase activity, respectively. Moreover, the binding of these molecules is accompanied by a conformational remodeling of TGase 2 [10,13].

**Figure 1:**
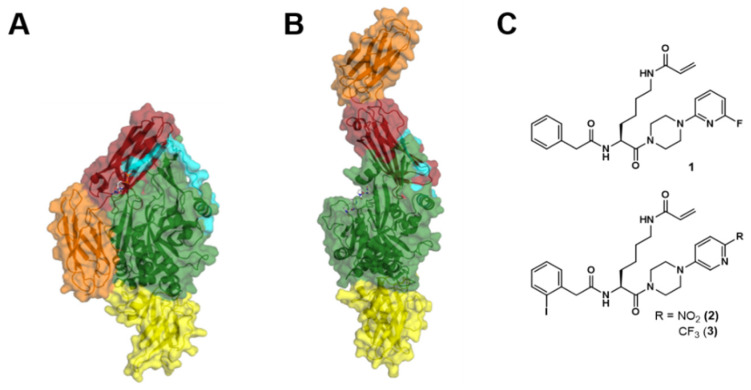
Crystal structures of human (h)TGase 2 in complex with GDP ((**A**), “closed” conformation) and with the irreversible inhibitor Ac-Pro-DON-Leu-Pro-Phe-NH_2_ ((**B**), “open” conformation) are shown as ribbon diagrams with a transparent surface. The N-terminal β-sandwich is colored in yellow, the α/β-catalytic domain in green, the flexible loop in turquoise, and the C-terminal β-barrels in red and orange. The figure was prepared using PyMOL (DeLano, W.L. The PyMOL Molecular Graphics System. Version 1.8 Schrödinger, LLC) and the PDB data 1KV3 [15] (**A**) and 2Q3Z [17] (**B**). Structures of *N*^ε^-acryloyllysine piperazides **1**–**3** (**C**).

TGase 2 is a monomeric protein and consists of 687 amino acids with a molar mass of 77 kDa [14]. The structure of TGase 2 is composed of four different domains. The N-terminal β-sandwich (amino acid residues 1–139) is followed by the α/β-catalytic core (amino acid residues 140–454), which harbors the catalytic triad for the transamidase activity (Cys-277, His-335, and Asp-358). The structure is completed by two consecutive β-barrels (amino acid residues 479–585 and 586–687). X-ray crystal structures of nucleotide-bound (GDP, GTP, ATP) TGase 2 reveal a compact (“closed”) conformation of TGase 2 in which the C-terminal β-barrels are folded in front of the α/β-catalytic domain (Figure 1A) [15,16]. In contrast to the nucleotide-bound TGase 2, X-ray crystal structures of TGase 2 in complex with irreversibly binding peptidic inhibitors reveal an elongated (“open”) conformation in which the C-terminal β-barrels do not mask the entry to the active site of the transamidase activity (Figure 1B) [17]. A flexible loop (amino acid residues 455–478) between the α/β-catalytic core and the subsequent β-barrel is considered the hinge region, which allows for the movement of the β-barrels [15,17,18,19]. Even though crystallization of the inhibitor-bound TGase 2 was performed in the presence of Ca^2+^, as it is required for binding the transamidase-directed compounds, no Ca^2+^ is found in the inhibitor-bound structures. Therefore, it is often assumed but speculative whether the “open” conformation is a representative snapshot of the Ca^2+^-activated conformation (or an ensemble of them). In this context, already before the first crystal structures of TGase 2 were solved, the group of Carlo Bergamini performed small-angle radiation scattering (SAXS and SANS) and Monte Carlo experiments based on a homology model of factor XIIIa (another member of the TGase family). Their results suggested that the binding of Ca^2+^ leads to a minimal rotation of the C-terminal β-barrels which uncovers the active site [18,19]. Similarly, Di Venere et al. suggested, based on results from spectroscopical techniques (CD, steady-state, and dynamic fluorescence), that Ca^2+^ induces an “opening” of the TGase 2 structure [20].

In addition to crystallization, radiation scattering, spectroscopic, and simulation methods, the conformational change of TGase 2 was also assessed by various other methods [21]. These included electrophoretic methods, such as native (nondenaturing) gel electrophoresis [17,22,23,24,25,26,27], kinetic capillary electrophoresis [28,29], and hydrogen/deuterium exchange with monitoring by mass spectrometry (HDX-MS) [30]. While these methods mainly focused on studying isolated TGase 2 and the conformational effects of various ligands, Förster resonance energy transfer-based assays were applied to study the conformational dynamics of TGase 2 in living cells [31,32]. All the mentioned studies proved that a significant conformational remodeling of TGase 2 occurs between nucleotide-bound forms and Ca^2+^-activated or inhibitor-bound forms.

Due to the intracellular concentrations of GTP (≈100–150 µM) and Ca^2+^ (≈0.1 µM), TGase 2 adopts conformations related to the “closed” conformation inside the cell, and thus, is considered to be largely transamidase-inactive under physiological conditions [31,32,33]. A conformational remodeling toward the “open” conformation occurs under physiological stress situations associated with a loss in Ca^2+^ homeostasis; in fact, there is an increase in the Ca^2+^ level, and the protein becomes transamidase-active [31,32]. While the transamidase activity of TGase 2, which is mainly exerted in the extracellular milieu, has been assigned to pathophysiological processes, such as fibrosis [34,35,36,37] and celiac disease [38,39], its GTP-binding ability is dominantly discussed in the context of tumor development and progression [40]. Various tumor entities are characterized by an increased TGase 2 expression, which is connected to poor prognosis [40]. Moreover, TGase 2 appears to be crucial for the survival of cancer stem cells [41,42,43,44]. This renders TGase 2 an attractive tumor-associated target for theranostic approaches with the primary focus on transamidase activity-directed covalent inhibitors, which induce a conformational remodeling of TGase 2 toward the “open” conformation. By the action of these agents, the transamidase activity is inhibited, and the binding of GTP is blocked [45,46,47]. Furthermore, it has been suggested that this conformational remodeling per se is cytotoxic [48,49]. Therefore, it is important to assess the effect of novel compounds on the conformational dynamics of TGase 2.

**Figure 2:**
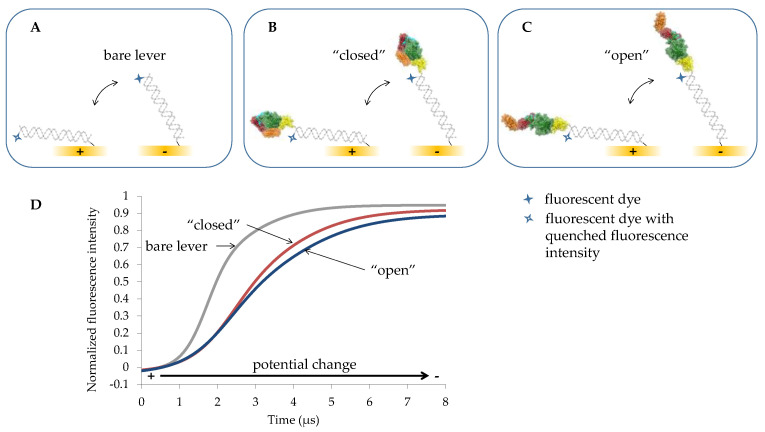
The switchSENSE^®^ principle to quantify conformational changes of TGase 2. The bare lever of double-stranded DNA of 48 base pairs length (**A**) is attracted by a positive potential, and the fluorescence intensity of the fluorescent dye is quenched due to the proximity to the gold electrode. Vice versa, the 16.3 nm-long lever is repelled by a negative potential, causing recovery of the fluorescence intensity (**D**). The lever with covalently bound TGase 2 in the “closed” conformation, with an extension of roughly 10 nm (**B**), will show increased friction during the upward motion, and consequently, slower recovery of the fluorescence intensity. An even higher friction is expected for the TGase 2 in the “open” conformation with an overall extension of roughly 15 nm (**C**). Accordingly, the maximum slope in the kinetics of the increasing fluorescence intensity can be used to quantify the extent of conformational change of the TGase 2 (**D**). For an accurate quantification, the slope in the kinetics of the bare lever is used as reference value.

An emerging technology for such assessment is switchSENSE^®^, illustrated in Figure 2. This dynamic method is based on electrically actuated DNA levers on gold electrodes combined with highly time-resolved fluorescence detection. Aside from their polyanionic nature, the levers ideally should have the greatest possible mechanical stiffness. Therefore, in most cases, double-stranded (ds) DNA [50,51,52], and in some cases, in particular when heavier proteins have to be actuated, even stiffer DNA origami structures are used as levers [53,54]. To prepare such actuators on surfaces, gold electrodes are functionalized with thiol-modified single-stranded (ss) DNA oligonucleotides. Each one of these ssDNA strands bears a fluorophore at the end opposite to its thiol moiety. In the present study, in the first step, a 48-base-pairs-long dsDNA lever is formed by hybridization of the surface-bound oligonucleotide with a complementary ssDNA strand (Figure 2A). Then, the motion of the resulting 16.3 nm-long, bare dsDNA levers is recorded by electrically triggered time-correlated single photon counting (E-TCSPC). This dynamic mode enables the detection of the fluorescence intensity with a time resolution of 100 ns when the lever is attracted by or repelled from the electrode by applying positive or negative potentials to the electrode, respectively. The fluorescence intensity correlates with the distance of the fluorophore from the gold surface. Due to energy transfer, the fluorescence is continuously quenched with reducing distance between the fluorophore and gold surface [55].

After taking the reference values for the dynamics of the bare dsDNA lever motion, a subsequent dehybridization provides an electrode surface with ssDNA for the hybridization of a TGase 2-ssDNA conjugate. The resulting double-stranded lever with a covalently bound TGase 2 on its distal end is then investigated by switching the potential from positive to negative values (Figure 2B,C). Compared to the movement of the bare dsDNA lever, the movement of the TGase 2-dsDNA conjugate is slowed down due to the larger hydrodynamic flow resistance of the conjugate. To which extent the latter is changed depends on the actual conformation of the attached TGase 2. To quantify the extent of conformational change of TGase 2 caused by different effectors, herein, the maximum slope in the kinetics of the increasing fluorescence intensity (upward motion of the TGase 2-dsDNA conjugate) is used (Figure 2D). The “closed” TGase 2 conformation exhibits an overall extension of roughly 10 nm, whereas it is roughly 15 nm for the “open” conformation (Figure 1A,B). Thus, a faster dynamic is expected for the “closed” conformation.

In the so-called static mode of the switchSENSE^®^ technique, a constant electric field keeps the DNA levers upright relative to the gold electrode [56]. Under these conditions, the binding of an analyte might be detected by fluorescence proximity sensing. Namely, molecular interactions, as well as conformational changes close to the fluorophore, might change its local environment, and hence, the fluorescence signal can be enhanced or attenuated.

In the present study, we characterize one recently described (**1**, Figure 1C) and two new *N*^ε^-acryloyllysine-derived irreversible inhibitors (**2** and **3**, Figure 1C) of TGase 2 concerning their impact on the conformational dynamic using switchSENSE^®^. The applicability of this method for TGase 2 was recently demonstrated by Staffler et al. [57] with a primary focus on assessing the binding kinetics of GTP analogs. Herein, two different strategies for the conjugation of TGase 2 with oligonucleotides were studied (non-directed and His-tag-directed amine conjugation). Furthermore, the influence of DMSO (solvent for the stock solutions of **1**–**3**) and Ca^2+^ (necessary for transamidase-active TGase 2) on the bare dsDNA lever motion was assessed to find a solvent composition, which avoids artifacts due to the interaction with the levers. Concerning data analysis in the dynamic mode, a novel computational method is introduced, which includes fitting a double logistic function to the time-resolved, normalized fluorescence intensity. In addition to the switchSENSE^®^ technique, classical nondenaturing or native polyacrylamide gel electrophoresis (native PAGE) experiments were performed to investigate the impact of the inhibitors on the conformation of TGase 2 using a complementary method. Native PAGE allowed for separating different conformations of soluble TGase 2 (without further modifying the protein) and quantifying their portions in the presence of increasing concentrations of inhibitor **2**. The present study is part of our efforts to develop novel radiotracers for TGase 2 [58]. The ^18^F-labeled analog of **1** is a potential candidate for the functional characterization of tumor-associated TGase 2 by positron-emission tomography (PET). We have already proven its suitability as a probe for radiometric activity-based detection of the protein in cells, cell lysates, and tissue sections [59]. Compounds **2** and **3** are envisaged for radiolabeling with iodine-123 for in vivo imaging by single photon emission computed tomography (SPECT) [60,61].

## 2. Results and Discussion

### 2.1. Conjugation of hTGase 2 to ssDNA via Non-directed and His-tag-directed Amine Labeling

For the conjugation of hTGase 2 (purchased as His_6_-tagged protein) to ssDNA, two different covalent conjugation strategies were applied using commercially available coupling kits. The first strategy used ssDNA modified with an NHS ester to label hTGase 2 via its N-terminus or lysine side chains (denoted hereafter as “non-directed labeling”). For the second strategy, a DNA-templated protein conjugation approach was followed using a tris-NTA functionalized guiding DNA that directs the NHS-functionalized ssDNA close to the N-terminus of hTGase 2 (denoted hereafter as “His-tag-directed labeling”) [62]. The second strategy aims for a site-selective protein conjugate, in contrast to the random conjugation to any available amino group by the non-directed labeling. By means of both strategies, hTGase 2 was successfully coupled to ssDNA, and the protein–DNA complex was purified (Figure S1 in Supplementary Materials). The non-directed labeling, however, provided ssDNA-hTGase 2 conjugates in a higher yield than the His-tag-directed labeling (27% versus 11% yield for the purified conjugate based on the used hTGase 2 amount, respectively). It is worth noting that both types of labeling led to a similar hybridization efficiency of the ssDNA-hTGase 2 conjugate to the ssDNA-functionalized chip. After hybridization, in both cases, a clear difference in the time-dependent fluorescence signal during operation in the dynamic mode was obtained for the hTGase 2-bound dsDNA lever compared to the bare dsDNA lever (Figure 2), i.e., a slower upward motion of the DNA lever with the attached protein was observed (Figure S2 in Supplementary Materials).

### 2.2. Assessment of GTPγS and Ca^2+^ Binding to hTGase 2

**Figure 3:**
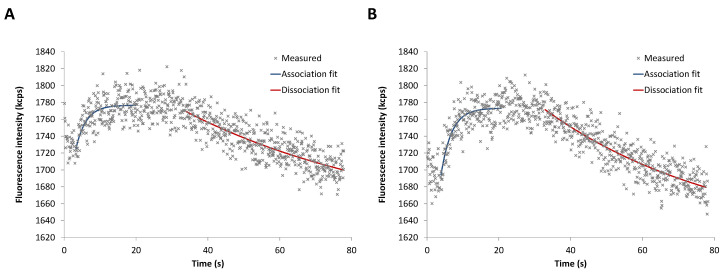
Fluorescence intensity versus time for the binding of GTPγS (20 nM) to hTGase 2-dsDNA lever (static mode) obtained by non-directed (**A**) or His-tag-directed (**B**) labeling of hTGase 2 to ssDNA.

To assess both the binding kinetics of GTPγS and the accompanied conformational changes of hTGase 2 for the two types of labeling, first, the static mode was applied. For this purpose, 20 nM of GTPγS were flushed over the electrodes. In the presence of GTPγS, the fluorescence signal increases (association, Figure 3); it decreases by removing the nucleotide (dissociation, Figure 3). The curve pattern is similar for the two hTGase 2-dsDNA levers. However, the His-tag-directed labeling (Figure 3B) provides a slightly higher fluorescence increase accompanied by a higher signal-to-noise ratio compared to the non-directed labeling (Figure 3A). Regression analyses according to Equations (5) and (8) (see Materials and Methods) gave the rate constants for the association and dissociation processes, summarized together with the dissociation constants, *K*_d_, in Table 1. Both labeling types resulted in comparable kinetic parameters, with *K*_d_ values in accordance with the value obtained by Staffler et al. under similar conditions [57].

Additionally, the binding of GTPγS and also Ca^2+^ to hTGase 2 should affect the upward motion of the hTGase 2-dsDNA lever in the dynamic mode. The size estimation of proteins conjugated to DNA levers is usually based on calculating the corresponding hydrodynamic diameter by considering both the switching motion of the protein–DNA conjugate and that of the bare DNA lever [56]. However, on the one hand, TGase 2 structures are known to be non-spherical, and on the other hand, an ensemble of conformations putatively contributes to the measured time-resolved fluorescence intensity. Consequently, the so-called “lollipop model” might not be most favorable for conformational change analysis. For TGase 2 in particular, Staffler et al. [57] reported distinct differences between their switchSENSE^®^ derived hydrodynamic diameters compared to values calculated from crystal structures and dynamic light scattering measurements. Therefore, a novel computational method was developed, including fitting a double logistic function to the time-resolved normalized fluorescence intensity. On this basis, the maximum slope at the inflection point of the fitted function was calculated. The inverse value of this slope is supposed to be a nearly proportional measure for the friction induced by structural changes in the conjugated protein or protein-inhibitor complex. This means the slower the upward motion of the DNA lever, the higher the inverse slope and, thus, the friction. In this context, we assume that the hydrodynamic friction of the conjugated enzyme depends on its conformation (Figure 2). It is expected that the “open” conformation (Figure 1B) will result in a higher friction than the “closed” one (Figure 1A). Consequently, the time-dependent normalized fluorescence intensity should show a bigger maximum slope and, thus, a smaller inverse slope for the “closed” conformation than for the “open” one (Figure 2D). Furthermore, it turned out that it is favorable to divide all inverse slopes within a series of conditions to be compared by the inverse slope of the bare DNA lever measured directly before the related consecutive experiments. The relative inverse slopes obtained in this way were then used to quantify conformational changes of the enzyme. Knezevic et al. [50] described a similar method by calculating the maximum of the first derivative of the time-dependent fluorescence curve smoothed by a fast Fourier transform filter. Nevertheless, there is some advantage of fitting a double logistic function to determine the inflection points. The herein used double logistic function is a sigmoidal function applicable to the “S”-shaped curves. This function is formed by a sum of two logistic functions [63]. With the help of double logistic functions, the complete, generally asymmetrical time-resolved curve of the normalized fluorescence intensity is sufficiently described. In particular, the region around the inflection point is reliably approximated. From the resulting explicit best-fit functions, derivative functions of arbitrary order (derived with respect to time) can be obtained to determine the inflection point with the maximum slope.

**Figure 4:**
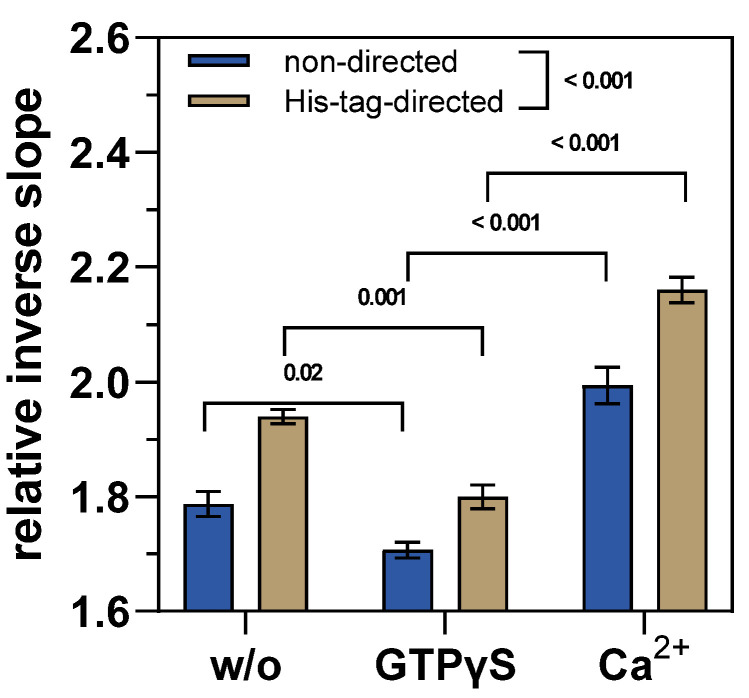
Changes of the hTGase 2-dsDNA lever motion as response to GTPγS (20 nM) and Ca^2+^ (1 mM) in dynamic mode for non-directed and His-tag-directed labeling of hTGase 2. The DNA lever motion was analyzed based on the inverse slope at the inflection point of the time-dependent fluorescence trace. The obtained values were divided by the inverse slope of the bare dsDNA. A higher relative inverse slope indicates higher friction of the hTGase 2-dsDNA lever. Shown are mean values (4 electrodes of one experiment) ± standard deviation. For statistical comparison of the relative inverse slopes within one labeling strategy and for a comparison of the labeling strategies in general, the values were subjected to a two-way repeated measures ANOVA with Tukey’s multiple comparison test (matched values for the relative inverse slopes obtained for the same electrode). *p* values are given above the brackets and values <0.05 were considered statistically significant. Although not shown, the relative inverse slopes for “w/o” and “Ca^2+^” are also significantly different with *p* = 0.008 (non-directed labeling) and *p* < 0.001 (His-tag-directed labeling).

Indeed, in the presence of GTPγS, the relative inverse slope is significantly smaller compared to the condition in pure buffer without GTPγS (Figure 4) since GTP analogs are known to shift the conformational dynamics of TGase 2 toward a “closed” conformation (Figure 1A). The subsequent removal of GTPγS and exposure to Ca^2+^ increases the relative inverse slope, even beyond the value of the hTGase 2-dsDNA lever in the absence of any effector (Figure 4; for kinetics, see Figure S2 in Supplementary Materials). This, in turn, is in accordance with recent data showing that the binding of Ca^2+^ favors a rather elongated conformation of TGase 2 [18,19,20,29,32], which should increase the friction of the DNA lever (Figure 2B). In this context, the conformational remodeling of TGase 2 might be a dynamic equilibrium of various intermediate conformations, which contribute to the friction of the hTGase 2-dsDNA lever. Therefore, in the absence of any effector, the observed friction is presumably of intermediate strength as no extreme conformational state is preferred.

By comparing the results for the different types of labeling, it becomes evident that the His-tag-directed labeling yields significantly (*p* < 0.001) higher values for the relative inverse slopes (Figure 4). This is in line with the results obtained for the binding kinetics of GTPγS, which were assessed in the static mode (Figure 3). Therefore, the site-selective coupling of TGase 2 to ssDNA (here via His-tag-guiding DNA) has a positive impact on the data quality for both measuring modes of switchSENSE^®^, as was recently also suggested by Staffler et al. [57]. In particular, for TGase 2, functionalization close to the N-terminus is favorable as the N-terminal β-sandwich is less involved in the conformational remodeling upon stimulation with GTP analogs and Ca^2+^. However, as mentioned above, the major drawback of the His-tag-directed labeling is the low yield during preparation of the conjugate. Therefore, we decided to perform the experiments for the conformational influence of the inhibitors **1**–**3** with the hTGase 2-ssDNA obtained by using non-directed labeling.

### 2.3. Assessment of the Binding of Inhibitors ***1***–***3*** to hTGase 2

Before the analysis of inhibitors **1**–**3** toward their impact on the conformational dynamics of TGase 2, their inhibitory potency had to be assessed. While the excellent inhibitory potency of compound **1** was previously shown [64], compounds **2** and **3** represent new compounds. The kinetic inhibitory potency of **2** and **3** was determined using a fluorimetric assay [65], revealing *k*_inact_/*K*_I_ values of 8340 M^−1^s^−1^ and 4500 M^−1^s^−1^, respectively (Table 2). Furthermore, IC_50_ values for the inhibition of transamidase activity were determined with a fluorescence anisotropy (FA) assay after 5 min of pre-incubation of protein and inhibitors (154 and 280 nM for **2** and **3**, respectively, Table 2) [64,66]. For the SDS-PAGE and *native (GTP-)PAGE* experiments, we used self-produced Twin-Strep-tagged hTGase 2, inhibited by compounds **1**–**3** with IC_50_ values in the range of those found toward the commercially obtained His-tagged hTGase 2 (Table 2, Figure S3 in Supplementary Materials).

**Figure 5:**
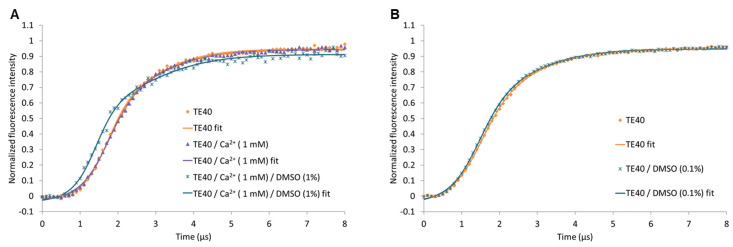
Influence of Ca^2+^ and DMSO on the upward motion of the hTGase 2-dsDNA lever (non-directed labeling). (**A**) Time-dependent normalized fluorescence intensity in TE40 buffer with or without 1 mM CaCl_2_ or with both 1 mM CaCl_2_ and 1% DMSO are shown. (**B**) Effect of reducing the DMSO concentration to 0.1%. Curves with the best-fit parameters (referred to as “fit”) of a double logistic function are shown for each solvent composition.

Due to limited solubility, the stock solutions of inhibitors **1**–**3** were usually prepared in DMSO, resulting in final DMSO concentrations for biochemical and biological assays between 0.1% and 5%. To determine potential interferences of DMSO, we evaluated the upward motion of the bare DNA lever using different solvent compositions (Figure 5). While Ca^2+^ at an applied concentration of 1 mM does not alter the shape of the time-dependent fluorescence curve, 1% DMSO leads to a left-shifted curve compared to pure aqueous conditions (Figure 5A). Fortunately, the shape of the curve is basically the same as that in aqueous condition when the DMSO concentration is lowered to 0.1% (Figure 5B). Therefore, the final DMSO concentration was set to 0.1% for all switchSENSE^®^ experiments with the inhibitors **1**–**3**. The reasons for the observed effects of DMSO cannot be given at this stage, but the results highlight the necessity to carefully evaluate the solvent composition to avoid measurement artifacts.

**Figure 6:**
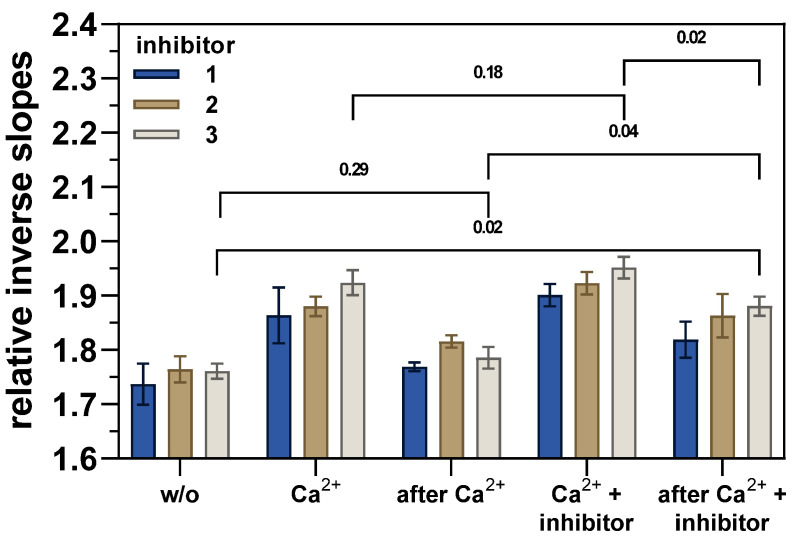
Changes of the hTGase 2-dsDNA lever upward motion (non-directed labeling) in response to Ca^2+^ (1 mM), the inhibitor (10 µM), and the removal of both effectors (dynamic mode, 0.1% DMSO). The notations for the resulting solvent composition and/or state of the hTGase 2-dsDNA lever are based on the treatment sequence explained in the text. The DNA lever motion was analyzed based on the inverse slope at the inflection point of the time-dependent normalized fluorescence intensity curve. The obtained values were divided by the inverse slope of the bare dsDNA to calculate respective relative values. A higher relative inverse slope indicates higher friction of the hTGase 2-dsDNA lever. Shown are mean values (four electrodes of one experiment) ± standard deviation. For statistical comparison of the effect of the three inhibitors and the treatment on the relative inverse slopes, the values were subjected to a two-way repeated measure ANOVA with Tukey’s multiple comparison test (matched values for the relative inverse slopes obtained for the same electrode). *p* values are given above the brackets and values <0.05 were considered statistically significant. The results of the multiple comparison test for inhibitor **3** are shown. For statistical comparisons of inhibitors **1** and **2**, see Figure S4 in Supplementary Materials.

After verification of their inhibitory activity and finding a proper solvent composition, the conformational impact of compounds **1**–**3** on hTGase 2 was assessed. For this purpose, the following treatment sequence was applied. (The notations for the resulting solvent composition and/or state of the hTGase 2-dsDNA lever used in Figure 6 are given in quotation marks.)

1. Pure TE40 buffer (“w/o”);
2. TE40 with 1 mM Ca^2+^ (“Ca^2+^”);
3. Pure TE40 buffer (removal of Ca^2+^; “after Ca^2+^”);
4. TE40 with 1 mM Ca^2+^ and 10 µM inhibitor (“Ca^2+^ + inhibitor”);
5. Pure TE40 (removal of Ca^2+^ and inhibitor; “after Ca^2+^ + inhibitor”).

The effects on the upward motion of the hTGase 2-dsDNA lever in terms of the relative inverse slope are shown in Figure 6.

As already described (Figure 4), the addition of Ca^2+^ increases the friction, and thus, the relative inverse slope in the curves of time-dependent normalized fluorescence intensity of the hTGase 2-dsDNA lever, which is decreasing after the removal of Ca^2+^ to almost the same level as before the addition of Ca^2+^ (differences are not statistically significant, Figure 6 and Figure S4 in Supplementary Materials). The simultaneous addition of Ca^2+^ and inhibitor seems to increase the friction to even higher values than Ca^2+^ alone (but this difference is not statistically significant for all inhibitors, Figure 6 and Figure S4 in Supplementary Materials). In case of inhibitor **3**, the subsequent removal of Ca^2+^ (“after Ca^2+^ + inhibitor”) decreases the friction to a level similar to Ca^2+^ alone (“Ca^2+^”) but still significantly higher compared to “w/o” and “after Ca^2+^” (Figure 6). For inhibitors **1** and **2**, this trend is similar; however, it should be noted that only some of the differences of “after Ca^2+^ + inhibitor” to “w/o” and “after Ca^2+^” are statistically significant (Figure S4 in Supplementary Materials). Consequently, the data might indicate that the three inhibitors shift the conformational equilibrium of hTGase 2 to more extended conformations upon their irreversible binding. Similar results were recently obtained by Staffler et al. [52] for the peptidic irreversible inhibitor Z-DON-Val-Pro-Leu-OMe (“Z-DON”) using switchSENSE^®^. Facing the rather slight changes in the relative inverse slopes between the different conditions and certain aging effects of the electrodes with increasing treatment cycles, it became necessary to use a complementary method to visualize the conformational changes of hTGase 2 due to the covalent or non-covalent binding of ligands.

### 2.4. Native (GTP-)PAGE Experiments to Asses the Conformational Change of hTGase 2 Induced by GTPγS, Ca^2+^, and the Inhibitors ***1***–***3***

In order to validate the results obtained by the switchSENSE^®^ method and to facilitate their interpretation, native PAGE was envisaged, a technique that has previously been used by several groups for studying the conformational dynamics of TGase 2 [17,26,27]. While switchSENSE^®^ can quantify a variety of individual conformations of an immobilized protein as an average value, native PAGE allows for separating soluble proteins in their individually folded conformation according to their molecular shape, size, and charge [67,68]. In contrast, the preparation of proteins for SDS-PAGE by heating the sample in the presence of SDS and a reducing agent, such as dithiothreitol (DTT) or 2-mercaptoethanol, leads to the unfolding of the soluble proteins and masking of their individual charge by binding to SDS. As a result, all proteins are rod-shaped with an approximately constant charge/mass ratio and, thus, are separated during SDS-PAGE solely by their molecular mass [69]. Both *native (GTP-)PAGE* and SDS-PAGE require relatively high protein concentrations in the µM range, which exceed the amount of protein immobilized for switchSENSE^®^ [70]. A detailed report on the conditions of our *native (GTP-)PAGE* and accompanying SDS-PAGE experiments can be found in Section 2.4 (see above) and in the Supplementary Materials (Discussion S1, Figure S5).

**Figure 7:**
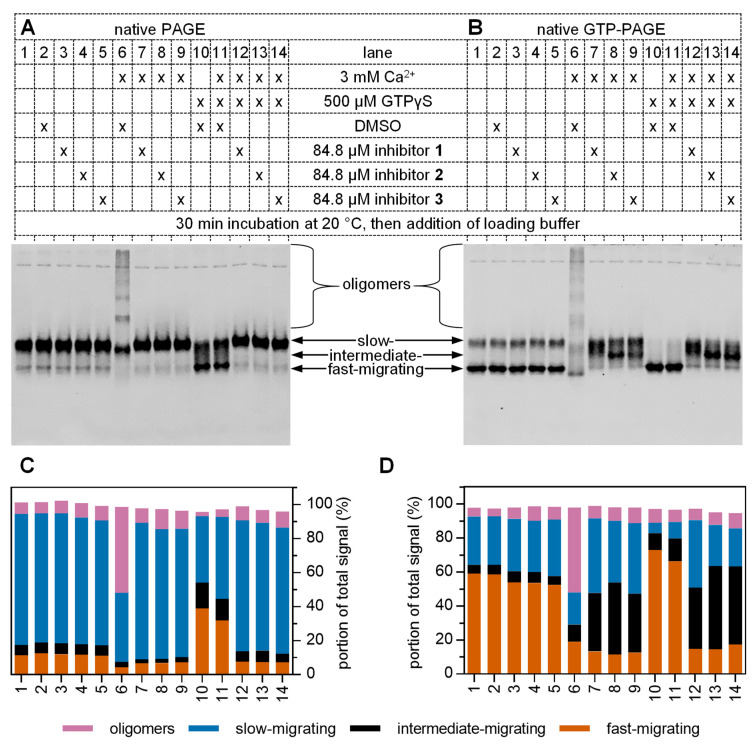
Influence of inhibitors **1**–**3** on the activity and conformation of hTGase 2 in the absence and presence of Ca^2+^ or/and GTPγS. *Native (GTP-)PAGE* was performed on 7% separating gels. The gel and the running buffer contained either no GTP (**A**) or 50 µM GTP (**B**). Samples of 8.23 µM self-produced hTGase 2 were incubated for 30 min at 20 °C in 100 mM MOPS pH 8.0, 100 mM NaCl, 5.33% (*v*/*v*) glycerol with CaCl_2_, DMSO or inhibitors **1**–**3** (2.12% DMSO), and GTPγS as indicated. After adding native loading buffer, 6 µL of each sample containing 3 µg of hTGase 2 was loaded onto the gel. After the electrophoretic separation, gels were stained with Coomassie Brilliant Blue G250, scanned, and the protein bands were quantified (Figure S6 in Supplementary Materials). Four species of hTGase 2, i.e., oligomers and slow-, intermediate-, and fast-migrating species, were identified. (**C**,**D**) Percentage of the signals of the four species per lane in (**A**,**B**), respectively, are plotted as stacked bar charts.

Analysis of hTGase 2 by *native PAGE* in the absence of any effector revealed the presence of an intense and a weak band, which can be assigned as slowly and fast migrating hTGase 2 species, respectively (Figure 7A, lanes 1, 2). By performing the *native PAGE* experiment after pre-incubation of hTGase 2 with 500 µM GTPγS (Figure 7A, lane 10), both bands showed a comparable intensity, indicating that the binding of GTPγS favors the fast migrating hTGase 2 species. This is comprehensible as this species was previously reported to be in compact [26] or “closed” [71,72] conformation. In this context, Begg et al. [26] demonstrated by native PAGE experiments with various TGase 2 mutants that the binding of GTP (or GTPγS) neutralizes the effect of an arginine residue within the GTP binding pocket that prevents the adoption of a compact (fast-migrating) species. While pre-incubation with 500 µM of GTPγS increased the portion of fast-migrating hTGase 2 species even in the presence of 3 mM of Ca^2+^ (Figure 7A–D, lanes 2 vs. 10, 11), an almost complete conformational change was only observed in the *native GTP-PAGE* (50 µM GTP present in the gel and the running buffer, Figure 7A vs. B, lanes 10, 11). This can be rationalized based on the fact that GTPγS (and GTP) dissociates from hTGase 2 during the electrophoretic separation. The presence of a high GTP concentration during that process favors a guanine nucleotide-bound hTGase 2. The observed change toward a fast-migrating species is consistent with the results obtained by switchSENSE^®^, where the friction and, thus, the relative inverse slope is lowered upon GTPγS binding (Figure 4). Therefore, both methods clearly indicate the conformational influence of GTPγS binding to hTGase 2.

In contrast to switchSENSE^®^, the conformational influence of Ca^2+^ on hTGase 2 could not reliably be assessed by *native (GTP-)PAGE*, as the addition of Ca^2+^ to the samples led to the self-catalyzed multimerization of hTGase 2 monomers by inter-crosslinking (Figure S6C, lane 6 in the Supplementary Materials), resulting in a ladder pattern with concomitant reduction of the monomer signals (Figure 7A,B, lanes 2 vs. 6 each) [27]. Nevertheless, we observed a slight shift in electrophoretic mobility for both the slow- and the fast-migrating monomeric species. Such multimerization was prevented in switchSENSE^®^ experiments due to the immobilization of monomeric hTGase 2.

The binding of inhibitors **1**–**3** and their conformational influence on hTGase 2 could not easily be deduced from *native PAGE* experiments as a similar band pattern was obtained in comparison to that for the incubation mixture of hTGase 2 without any effector (Figure 7A, lanes 1, 2 vs. 3–5, 7–9). A hint for inhibitor binding is the lowered portion of the fast-migrating hTGase 2 species in the presence of Ca^2+^ (Figure 7A, lanes 1–5 vs. 7–9). By forcing a more significant portion of hTGase 2 into the fast-migrating species due to the addition of 500 µM of GTPγS to the incubation mixture (Figure 7A, lanes 6 vs. 11), the effect of inhibitors **1**–**3** on preventing the formation of the compact hTGase 2 conformation became more apparent (Figure 7A, lanes 11 vs. 12–14). This principle was refined in the *native GTP-PAGE,* where the presence of GTP in both the gel and the running buffer resulted in a shift of the majority of hTGase 2 to the compact, fast-migrating conformation (even without the addition of GTPγS to the incubation mixture). This allowed for a much clearer visualization of compounds’ **1**–**3** inhibitory effect on this conformational shift (Figure 7B, lanes 1/2 vs. 7–9). The observed behavior of inhibitors **1**–**3** is in line with results previously shown by Pinkas et al. [17], Stamnaes et al. [71], and Gundemir et al. [72] for other active site-directed irreversible inhibitors. As expected, the presence of 3 mM Ca^2+^ was required for inhibitors’ **1**–**3** effect on hTGase 2 conformation (Figure 7B, lanes 3–5 vs. 7–9), and the additional presence of 500 µM GTPγS did not prevent the binding of the inhibitors (Figure 7B, lanes 3–5 vs. 7–9 vs. 12–14). By comparing the results of the *native PAGE* and *native GTP-PAGE* experiments, it becomes evident that the presence of GTP in the gels and the running buffer and, thus, the initial shift of the majority of protein molecules to the closed conformation was crucial for detecting effects of inhibitor binding on the conformational dynamics of hTGase 2.

In *native PAGE* experiments, inhibitor-bound hTGase 2 cannot be distinguished from sole hTGase 2 as both appear as slow migrating species. This is in accordance with results previously shown by Pinkas et al. [17] for Ac-P(DON)LPF-NH_2_. However, our switchSENSE^®^ results revealed a difference in the friction for the upward motion of the hTGase 2-dsDNA lever in the absence and presence of inhibitors **1**–**3**, meaning conformational remodeling occurs upon inhibitor binding, which cannot be resolved by *native PAGE*.

**Figure 8:**
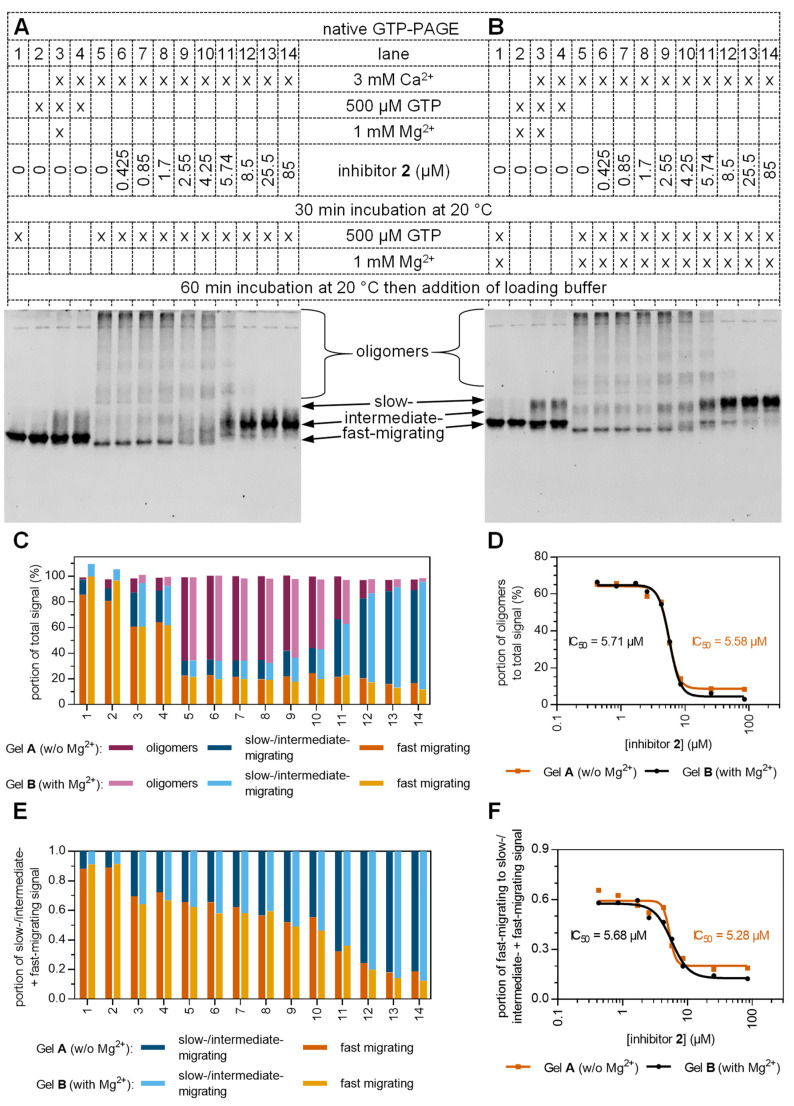
Effect of increasing concentrations of inhibitor **2** on the activity and the conformation of hTGase 2. *Native GTP-PAGE* was performed on 7% separating gels. Both the gel and the running buffer contained 50 µM GTP without (**A**) and with an additional 500 µM MgCl_2_ (**B**). Samples of 8.23 µM self-produced hTGase 2 were first incubated for 30 min at 20 °C in 100 mM MOPS pH 8.0, 100 mM NaCl, 2.12% (*v*/*v*) DMSO, 5.33% (*v*/*v*) glycerol with CaCl_2_, GTP, MgCl_2_, and different concentrations of inhibitor **2**, as indicated. GTP and/or MgCl_2_ were added as indicated, and all samples were incubated for 60 min at 20 °C. After adding native loading buffer, 6 µL of each sample containing 3 µg of hTGase 2 was loaded onto the gel. After electrophoretic separation, gels were stained with Coomassie Brilliant Blue G250, scanned, and the protein bands were quantified (Figure S7 in Supplementary Materials). Four species of hTGase 2, i.e., oligomers and slow-, intermediate-, and fast-migrating species, were identified, with slow- and intermediate-migrating species combined for the quantitative analysis. (**C**) Percentage of the signals of the three species per lane and (**D**) percentage of the oligomers’ signal plotted versus the concentration of inhibitor 2. (**E**) Signals of the slow-/intermediate-migrating and fast-migrating species were added and set to one. (**F**) Portion of the fast-migrating species in (**E**) was plotted against the concentration of inhibitor 2. The IC_50_ values in (**D**,**F**) were calculated by non-linear regression according to Equation (S29) in Supplementary Materials.

In *native GTP-PAGE* experiments, inhibitor binding led to the formation of an intermediate hTGase 2 species, which was more pronounced when samples were also pre-incubated with 500 µM of GTPγS (Figure 7B,D, lanes 7–9 vs. 12–14). These intermediately migrating species are hypothesized to represent hTGase 2 monomers (net charge of -37.8 at a pH of 8.3, calculated from the amino acid sequence of in-house produced N-terminally Twin-Strep-tagged hTGase 2 shown in Figure S8 in Supplementary Materials) that have bound inhibitors **1**, **2**, or **3** and simultaneously interact with guanosine nucleotide triphosphates that are mostly quadruple-negatively charged (pK values of H_3_GTP^2−^ (N_7_^+^H), H_2_GTP^3−^ (γP-OH), and HGTP^4−^ (N_1_H) are 2.9, 6.5, and 9.6 [73,74]), with the negative charges of the latter ligands leading to higher electrophoretic mobility. It should be pointed out that this effect occurred in the absence of Mg^2+^, which was neither included in the incubation mixture nor the gel and the running buffer by that time as it is not mandatory for the binding of GTP or GDP to hTGase 2 [15,75,76]. However, reported native PAGE experiments investigating the effects of guanosine nucleotides on TGase 2 conformation were typically performed in the presence of Mg^2+^ [26,72,77]_._ To further support our hypothesis on the binding of guanosine nucleotide triphosphates to hTGase 2 and investigate the effect of Mg^2+^, we determined the electrophoretic behavior of hTGase 2 by *native GTP-PAGE* when incubated with increasing concentrations (0–85 µM) of inhibitor **2** in the absence and presence of Mg^2+^ (Figure 8). We speculated that in the presence of Mg^2+^, some of the negative charges of GTP complexed with inhibitor-bound hTGase 2 would be masked, resulting in electrophoretic shifts similar to that of unbound hTGase 2 (Figure S5B in Supplementary Materials, lanes 1, 2 vs. 4–9). Analyzing the lanes of the *native GTP-PAGE* without Mg^2+^ (Figure 8A,C) revealed the presence of increasing amounts of the monomeric intermediate-migrating species with a concomitant decrease in hTGase 2 oligomers when increasing the concentration of **2**. In the presence of Mg^2+^ (Figure 8B,C), this decrease in oligomeric signal was almost identical, leading to similar dose-response curves and IC_50_ values for inhibitor **2** when quantifying the portion of hTGase 2 oligomers to the total signal (Figure 8D). However, in the *native GTP-PAGE* with Mg^2+^, we observed increased amounts of the slow-migrating instead of the intermediate-migrating hTGase 2 species, supporting our hypothesis that Mg^2+^ decreases the electrophoretic mobility of inhibitor-hTGase 2 complexes bound to guanosine nucleotide triphosphates. To show that Mg^2+^ does not affect the conformational remodeling of the hTGase 2 upon binding to inhibitor **2**, we determined the portion of the signal of the closed conformation to the signals of all monomeric hTGase 2 species (Figure 8E,F). The obtained IC_50_ values were independent of the presence of Mg^2+^ and almost identical to the values for the inhibition of hTGase 2, shown in Figure 8D. This highlights that the binding of inhibitor **2** is accompanied by functional inhibition and conformational remodeling. Our results illustrate the importance of Mg^2+^ to obtain good separation and resolution of the conformational species of hTGase 2 in *native GTP-PAGE* experiments, as particularly shown in Figure 8A vs. B, lanes 3 and 4.

Comparing the inhibition constants obtained for compound **2** by *native GTP-PAGE* with those IC_50_ values determined in the FA assay after pre-incubation of enzyme and inhibitor for 30 min (35–47 nM, Figure S3B in Supplementary Materials), there is a difference of approx. two orders of magnitude mainly due to the higher hTGase 2 concentration in the *native GTP-PAGE* experiments (62–64 nM, FA assay vs. 8.23 µM, *native GTP-PAGE*). On the other hand, calculating the ratio of the IC_50_ and the molar enzyme concentration resulted in similar values for the two methods (0.57–0.74 (see Figure S3 in Supplementary Materials) vs. 0.64–0.69 (calculated from values in Figure 8D,F)).

Considering the crystal structures of guanine nucleotide-bound and inhibitor-bound TGase 2 (Ac-Pro-DON-Leu-Pro-Phe-NH_2_, Figure 1A) and in silico structure analyses [46], our hypothesis that inhibitors **1**-, **2**-, or **3**-bound hTGase 2 are still able to bind GTP at the original binding pocket of guanine nucleotides appears unlikely. However, one could speculate that the guanine nucleotide binding site on inhibitor-bound TGase 2 might differ from that original binding pocket and/or that the interaction might be of low affinity and thus is only detectable due to the high concentration of GTP in the running buffer and the gel (50 µM). Further experiments are needed to prove our hypothesis ultimately.

**Figure 9:**
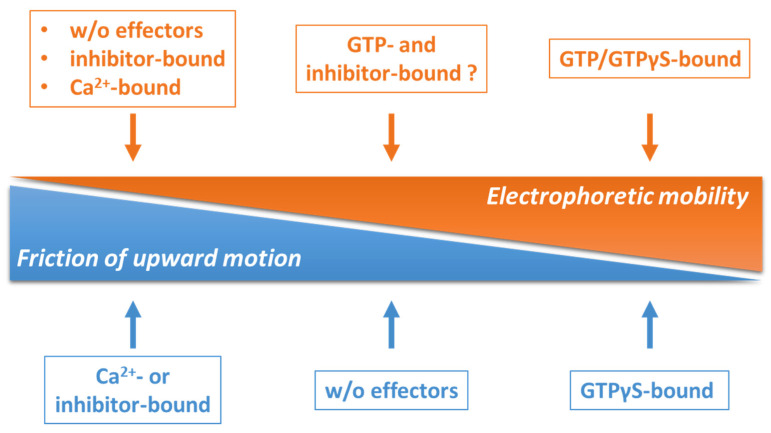
Qualitative comparison of the results on the conformational dynamics of hTGase 2 obtained by switchSENSE^®^ and *native (GTP-)PAGE*. For switchSENSE^®^, the friction of the upward motion was measured (blue, decreases from left to right). In contrast, for *native (GTP-)PAGE*, the electrophoretic mobility (orange, increases from left to the right) was assessed.

A summary by means of a qualitative side-by-side comparison between the results obtained by switchSENSE^®^ and *native (GTP-)PAGE* is shown in Figure 9.

## 3. Conclusions

We herein characterize the conformational impact of the three *N*^ε^-acryloyllysine piperazides **1**–**3** on the enzyme hTGase 2. These inhibitors bind irreversibly to hTGase 2. Two complementary biophysical techniques were used for this study: switchSENSE^®^ and *native (GTP-)PAGE*. SwitchSENSE^®^ allowed for a direct assessment of the conformational remodeling of hTGase 2 by inhibitors **1**–**3** by providing an average value for a variety of hTGase 2 conformations. The results are in accordance with the general presumption that active-site directed irreversible inhibitors lead to a conformational remodeling toward an extended conformation. These results are supported by *native GTP-PAGE* experiments, in which the binding and conformational impact of inhibitors **1**–**3** were investigated more indirectly as hTGase 2 was initially forced into a compact conformation by adding guanosine nucleotides to the gel and the running buffer. The binding of inhibitors to the active site led to a conformational remodeling in a concentration-dependent manner, which was detectable as a shift in the electrophoretic mobility. Moreover, this method could conflate the conformational remodeling upon inhibitor binding to the enzyme’s activity.

## 4. Materials and Methods

### 4.1. Materials

Magnesium chloride hexahydrate (≥99%), TWEEN 20 (Ph. Eur.), TRIS^®^ (≥99.9%), MOPS (≥99.5%), EDTA (>99%), EGTA (≥99%), glycine (≥99%), GTP trihydrate disodium salt (≥90%), acrylamide-bisacrylamide solution (ROTIPHORESE^®^ Gel 40 (37.5:1)), ammonium peroxydisulphate (≥98%), TEMED (≥99%), SDS (≥99%), 2-mercaptoethanol (≥99%), Coomassie Brilliant Blue G250 sodium salt, and HCl (37%, p.a.) were commercially available from Carl Roth. *N*,*N*-Dimethylated casein from bovine milk (DMC, C9801-5G, lot SLCH5943; ≥90% by colorimetric assay) was obtained from Sigma-Aldrich. Sodium chloride (≥99.5%) was purchased from VWR and Carl Roth. Calcium chloride dihydrate (>99.5%) was obtained from Fluka and Sigma-Aldrich, TCEP (99%) was available from abcr, and DTT (>99.5%) from AppliChem. DMSO (>99.5%) and glycerol (≥99% by GC) were purchased from Merck. Recombinant His_6_-hTGase 2 (T022, lot 0716a; >90% by SDS-PAGE) was obtained from Zedira (Darmstadt, Germany), and GTPγS (free acid, ≥99% by HPLC) was purchased from Jena Bioscience. All chemicals were used without further purification. PageRuler™ Plus Prestained Protein was available from ThermoFisherScientific (Vilnius, Lithuania). Deionized water (κ~55 nS/cm) was purified with a MicroPure system (ThermoScientific) or an Astacus^2^ water purifier (membraPure). DNA oligonucleotides were available either from Dynamic Biosensors (Munich, Germany) or from biomers.net (Ulm, Germany). The enzyme conjugation kits without a directing agent (CK-NH2-1-B48) and with His-direction (PF-NH2-2-B48) as well as the used buffers (A, B, C, H), regeneration solution, passivation solution, and chips (MPC-48-1-R1) were purchased from Dynamic Biosensors.

### 4.2. Plasmid Generation and Bacterial Expression of hTGase 2

The gene of hTGase 2 (Uniprot ID: P21980) with an *N*-terminal thrombin cleavage site was cloned into the pPSG-IBA105 vector containing the sequence for an *N*-terminal Twin-Strep-tag (IBA Lifesciences GmbH, Göttingen, Germany) to obtain the final plasmid pPSG-IBA105/TCS-hTGase 2. The plasmid construct was verified by DNA sequencing (Eurofins Genomics Germany GmbH) and then transformed into *E. coli* BL21(DE3) by the heat–shock method [78,79]. hTGase 2-expressing *E. coli* cells were lysed by lysozyme treatment, and hTGase 2 was purified by affinity chromatography on an ÄKTA purifier UPC 10 FPLC equipped with a Strep-Tactin XT cartridge (GE Healthcare Europe GmbH, Freiburg, Germany). Identity and purity of self-produced hTGase 2 were verified by SDS-PAGE and Western Blot analysis, respectively. Protein concentration was determined according to Bradford [80]. More detailed descriptions of the generation and characterization of the final plasmid construct pPSG-IBA105/TCS-hTGase 2 and the expression, purification, and characterization of the self-produced hTGase 2 protein will be published elsewhere (manuscript in preparation).

### 4.3. switchSENSE^®^

#### 4.3.1. Preparation of Protein–DNA Conjugates

The conjugation of hTGase 2 to an ssDNA that can hybridize onto a chip was performed according to the switchSENSE^®^ supplier’s protocols: The kit CK-NH2-1-B48 uses a crosslinker (with *N*-hydroxysuccinimide (NHS) ester and a maleimide group) to couple an amine group of hTGase 2 to the thiol group of an ssDNA oligonucleotide. The kit PF-NH2-2-B48 uses the His-tag of hTGase 2 to couple a so-called guiding ssDNA carrying tris-NTA (three nitrilo-triacetic acid groups) [81]. This was followed by hybridizing the desired, crosslinker-activated ssDNA to the guiding ssDNA. After the coupling of hTGase 2 to the crosslinker-activated ssDNA, the guiding ssDNA was replaced from the crosslinker-activated ssDNA by hybridizing it to a replacement oligonucleotide. The hTGase 2-ssDNA conjugates were purified with a proFIRE (Dynamic Biosensors; Figure S1 in Supplementary Materials), and the concentration subsequently determined with a NanoDrop (Thermo Scientific, Waltham, MO, USA).

#### 4.3.2. Measurements

The switchSENSE^®^ experiments were performed with a DRX^2^ (Dynamic Biosensors, excitation 600–630 nm, emission 650–686 nm). All experiments were performed in TE40 (10 mM TRIS pH 7.4, 40 mM NaCl, 50 µM EDTA, 50 µM EGTA, 100 µM TCEP, 0.05% Tween 20) when not explicitly stated otherwise. For the dynamic mode measurements, the standard operation procedures as described in [56] were applied. For measurements of the kinetics of the interaction of both GTPγS and CaCl_2_ with hTGase 2, firstly, the ssDNA-hTGase 2 conjugate was hybridized for 360 s to the ssDNA capture strand on the gold microelectrodes of the chip. With 500 nM, the conjugate concentration was chosen relatively high to achieve full coverage of the electrodes. Secondly, 1000 µL of 20 nM GTPγS in TE40 were flushed over the electrodes, followed by about 4000 µL of TE40 (2000 µL/min), to obtain access to the association as well as the dissociation, respectively. Changes in the fluorescence intensity during the GTPγS interaction were recorded using fluorescence proximity sensing (static mode).

Prior to the measurement of the switching kinetics of the differently conjugated hTGase 2, a complementary ssDNA without the enzyme (500 nM) was hybridized to the single-stranded capture strands on the chip electrodes to obtain the reference kinetics of the dsDNA lever. In addition, to determine the impact of CaCl_2_ and DMSO on the movement of the bare dsDNA lever, and thus on reference kinetics, its switching behavior was investigated in TE40, TE40 + 1 mM CaCl_2_, TE40 + 1 mM CaCl_2_ + 1% DMSO or TE40 + 1 mM CaCl_2_ + 0.1% DMSO.

Then, the dehybridization of the reference lever occurred during a regeneration step followed by the hybridization with an ssDNA-hTGase 2 conjugate (500 nM). Thereafter, the switching kinetics was recorded. Moreover, either GTPγS (500 nM in TE40 buffer) or CaCl_2_ (1 mM in TE40 buffer) were flushed over the chip and left in the channel of the microelectrodes for the determination of the switching kinetics of the presumably “closed” or “open” conformation, respectively (Figure S2 in Supplementary Materials).

The impact on the hydrodynamic drag of the hTGase 2-functionalized lever was investigated under identical conditions for the three irreversible inhibitors **1**–**3**: At first, the switching kinetics of the dsDNA lever and the hTGase 2-dsDNA conjugates were recorded. Secondly, the switching kinetics of the hTGase 2-dsDNA conjugates during and after Ca^2+^ exposure were recorded using TE40 + 1 mM CaCl_2_ or TE40, respectively. Finally, the influence of the irreversible inhibitor on the switching kinetics of the hTGase 2-dsDNA conjugates was investigated. In this process, the hydrodynamic drag was analyzed during the exposure to 10 µM inhibitor (for 6 min) dissolved in TE40 + 1 mM CaCl_2_ + 0.1% DMSO and to TE40 afterward.

#### 4.3.3. Kinetics of GTPγS Interaction with hTGase 2 by Proximity Sensing in the Static Mode

The formation of the protein–ligand complex of GTPγS and hTGase 2 can be represented by the reaction equation expressed by Equation (1).

(1) $$P+L\;\begin{array}{c} k_{on}\\ ⇌\\ k_{off} \end{array}\;PL$$

In Equation (1), the protein, the ligand, and the protein–ligand complex are denoted by *P*, *L*, and *PL*, respectively. The rate constants of the formation and dissociation reactions are *k_on_* and *k_off_*, respectively.

##### Association

The concentration of the protein–ligand complex, [PL], at a certain time of association $t_{a}$ is ${\left[PL\right]}_{a}$. At the beginning of the association, there is no surface-bound complex present, i.e., [PL]=0, and the ligand concentration is assumed to be constant at $\left[L\right]=c_{GTP\gamma S}$. This neglects diffusion limitation by assuming continuous replacement of the medium at the electrode surface in the microfluidic channel. Thus, the time-dependent equilibrium fraction reached at the association time $t_{a}$ can be expressed by Equation (2). For derivation, see Equations (S1) to (S5) in Supplementary Materials.

(2) $$\frac{{\left[PL\right]}_{a}}{{\left[PL\right]}_{\infty }}=1-e^{-k_{a}\cdot t_{a}}$$

In Equation (2), ${\left[PL\right]}_{\infty }$ is the equilibrium concentration of the protein–ligand complex, and *k_a_* is the apparent rate constant in the association experiment, defined by Equation (3).

(3) $$k_{a}=c_{GTP\gamma S}\;k_{on}+k_{off}$$

The assumption needed to be applied for the fluorescence proximity sensing is given in Equation (4).

(4) $$\frac{F_{a}-F_{a,s}}{F_{a,e}-F_{a,s}}=\frac{{\left[PL\right]}_{a}}{{\left[PL\right]}_{\infty }}$$

Here, *F_a_*, *F_a,s_*, and *F_a,e_* are the fluorescence intensities at the association time *t_a_*, at the start, and when the equilibrium is reached, respectively. This means that the ratio of fluorescence intensity changes since the beginning of the association, $F_{a}-F_{a,s}$, to the change until the equilibrium is reached, $F_{a,e}-F_{a,s}$, is equal to the ratio of the amount of surface-bound protein–ligand complex to its ligand concentration-dependent equilibrium amount. Equations (4) and (2) finally lead to Equation (5), which describes the fluorescence intensity as a function of the association time *t_a_*.

(5) $$F_{a}=\left(F_{a,e}-F_{a,s}\right)\left(1-e^{-k_{a}\cdot t_{a}}\right)+F_{a,s}$$

Equations (4) and (5) are valid, notwithstanding whether the fluorescence intensity is enhanced or decreased by the ligand binding. The corresponding Jacobian matrix for the fitting procedure to determine $F_{a,e}$, $F_{a,s}$, and $k_{a}$ is given in Equations (S6) and (S6a–d).

##### Dissociation

During the dissociation, the concentration of the free ligand is assumed to be constantly zero. Consequently, Equation (6) should be valid (for derivation, see Equations (S7) to (S10) in Supplementary Materials).

(6) $$\frac{{\left[PL\right]}_{d}}{{\left[PL\right]}_{d,0}}=e^{-k_{off}\cdot t_{d}}$$

Equation (6) describes the dissociation time, $t_{d}$, dependent ratio of the amount of surface-bound protein–ligand complex at $t_{d}$, ${\left[PL\right]}_{d}$, to its amount at the beginning of the dissociation, ${\left[PL\right]}_{d,0}$. Analogously to the association, the fluorescence proximity sensing provides the fluorescence intensity $F_{d}$ at $t_{d}$. With the fluorescence intensity at the dissociation start, $F_{d,s}$, and at its extreme value for $t_{d}\to \infty$, $F_{d,e}$, the relation in Equation (7) is supposed to be valid.

(7) $$\frac{F_{d}-F_{d,e}}{F_{d,s}-F_{d,e}}=\frac{{\left[PL\right]}_{d}}{{\left[PL\right]}_{d,0}}$$

Combining Equations (6) and (7), the time-dependent fluorescence intensity during the dissociation is given by Equation (8).

(8) $$F_{d}=\left(F_{d,s}-F_{d,e}\right)\left(e^{-k_{off}\cdot t_{d}}\right)+F_{d,e}$$

The corresponding Jacobian matrix for the fitting procedure to determine $F_{d,e}$, $F_{d,s}$, and $k_{off}$ is given in Equations (S11) and (S11a–d).

##### Determination of *k*_*on*_ and *k*_*off*_

Equations (5) and (8) were fitted to the measured fluorescence intensities of the association and dissociation experiments with 20 nM GTPγS, respectively. This was performed by a damped Gauss–Newton algorithm (see Equations (S12) and (S13) in Supplementary Materials) to determine the best-fit parameters, and consequently, *k*_*on*_ and *k*_*off*_. The quotient *k*_*off*_/*k*_*on*_ is the dissociation constant, *K*_*d*_. Additionally, the standard errors of these parameters were estimated (Equations (S14) to (S21) in Supplementary Materials) [82,83].

#### 4.3.4. Detection of Conformational Changes in the Dynamic Mode

An empirical equation with seven parameters of the double logistic function was used to model the normalized fluorescence intensity as a function of time during the recovery of the fluorescence intensity during the upward motion (Figure 2). This double logistic function is formed by the sum of two logistic functions, as is shown in Equation (9) [63].

(9) $$y=y_{0}+A\left[\frac{p}{1+e^{\frac{x-x_{1}}{k_{1}}}}+\frac{1-p}{1+e^{\frac{x-x_{2}}{k_{2}}}}\right]$$

In Equation (9), y is the normalized fluorescence intensity at the time x, $y_{0}$ is the left asymptotic fluorescence intensity value, A is the difference between the right and the left asymptotic fluorescence intensity values, p (0≤p≤ 1) is the fraction of the first logistic function part, and 1−p is the fraction of the second. The time parameters, $x_{1}$ and $x_{2}$, are centers of these parts with $k_{1}$ and $k_{2}$ as their corresponding slope factors, respectively. The best-fit parameters were used to determine the inflection point by calculating the zero of the second derivative of this function with respect to time, where the absolute value of the first derivative is at its maximum (see Equations (S22) to (S26) in Supplementary Materials). Again, a damped Gauss–Newton algorithm was used to determine the best-fit parameters (for the corresponding Jacobian matrix see Equations (S27) and (S27a–g), and for the parameter vector see Equation (S28) in Supplementary Materials). At this point the reciprocal of the first derivative was calculated. This reciprocal maximal slope was calculated for four electrodes, and consequently, the mean and the standard deviation were obtained for each conformational state.

The free software GNU Octave [84] was used for programming routines needed to compute all rate constants, inflection points, and corresponding errors.

### 4.4. Polyacrylamide Gel Electrophoresis (SDS-PAGE and Native PAGE)

Sodium dodecyl sulfate-PAGE (SDS-PAGE) and native PAGE were performed on 4% stacking and 7% separating gels using Laemmli buffers [85] (60 min with 120 V, 300 mA and 30 W at room temperature) and the same buffers without SDS (80 min with 120 V, 50 mA and 30 W at 4 °C), respectively. Buffers and gels were as follows: (i) for SDS-PAGE: stacking gel: 0.125 M TRIS pH 6.8, 4% (*v*/*v*) acrylamide-bisacrylamide, 0.1% (*m*/*v*) SDS; separating gel: 0.375 M TRIS pH 8.8, 7% (*v*/*v*) acrylamide-bisacrylamide, 0.1% (*m*/*v*) SDS; running buffer: 0.025 M TRIS pH 8.3, 0.2 M glycine, 0.1% (*m*/*v*) SDS; (ii) for native PAGE: the same buffers and gels were used without the addition of SDS. Native PAGE experiments, where 50 µM of GTP was added to the stacking and separating gels and the running buffer, are specified as “*native GTP-PAGE*” (with italic letters). In contrast, experiments without GTP are denoted as “*native PAGE*” (with italics letters). The term “*native (GTP-)PAGE*” (with italics letters) is used when we refer to both methods. Further conditions of the SDS-PAGE and *native (GTP-)PAGE* are detailed in the legends of Figure 7 and Figure 8 and Figures S4–S6 (Supplementary Materials). For the loading of samples onto the stacking gel, three parts of the sample solution were mixed with one part of the respective loading buffer. In the case of SDS-PAGE, SDS loading buffer (0.24 M TRIS, 40% (*v*/*v*) glycerol, 8% (*m*/*v*) SDS, 2% (*v*/*v*) 2-mercaptoethanol, and 0.08% (*m*/*v*) bromphenol blue) were used, followed by the denaturation of the mixture for 5 min at 60 °C. For *native (GTP-)PAGE,* native loading buffer (0.24 M TRIS, 40% (*v*/*v*) glycerol, 0.08% (*m/v*) bromphenol blue) was added to the sample solution instead. The marker for SDS-PAGE was PageRuler™ Plus Prestained Protein Ladder. For *native (GTP-)PAGE*, an in-house produced Calmodulin derivative, mCherry-CaM_NL_, was used as a reference protein, whose preparation will be the content of a future report. After electrophoretic separation, gels were stained with Coomassie Brilliant Blue G250 according to Kang et al. [86] and destained several times with 10% ethanol containing 2% phosphoric acid. Afterward, the gels were scanned using a LI-COR Odyssey Fc Imager (700 channel) and analyzed using Image Studio software version 5.2.5. Detected bands were quantified based on the general procedure published by Wagstaff et al. [70] and as detailed in legends to Figures S5 and S6. For *native (GTP-)PAGE* experiments, the lane of the reference protein mCherry-CaM_NL_ was excluded from all images of the scanned gels before analysis.

### 4.5. Characterization of the Inhibitory Potency of Compounds ***1***–***3***

The kinetic characterization of compounds **2** and **3** by means of their *k*_inact_/*K*_I_ values was performed using a fluorimetric activity assay (substrate Z-Glu(HMC)-Gly-OH used at 35 µM and enzyme His_6_-hTGase 2 at 2 µg/mL) according to the literature [64,65,87]. The IC_50_ values of inhibitors **1**–**3** were determined by a fluorescence anisotropy (FA)-based assay as described recently [64,66] with some alterations, including a reduced total assay volume of 100 µL, a final concentration of 0.74 µM R-I-Cad, and DTT instead of TCEP as a reducing agent for hTGase 2. For the analysis of inhibitor **1** on self-produced hTGase 2, 2 µg/mL of the enzyme was used to guarantee comparability with the value reported by Wodtke et al. [64], whereas all other experiments were performed with 5 µg/mL of either commercial or self-produced hTGase 2 (corresponding to 64.1 and 61.6 nM, respectively, when assuming 100% active protein). Parallel and perpendicular fluorescence intensities were measured over 1190 s in 35 s intervals (**1**) or over 1776 s in 48 s intervals (**2**, **3**) and used to calculate the FA. Data of FA for the first 875 s (**1**) or 384 s (**2**, **3**) were analyzed by linear regression, with slopes corresponding to enzymatic rates. Rates in the presence of inhibitor were normalized by dividing them by the respective rate in the absence of inhibitor, and normalized rates were analyzed according to Equation (S29) in Supplementary Materials to calculate values of IC_50_. Data analysis was performed with Microsoft Excel (Microsoft Office Professional Plus 2016, Microsoft^®^ Corporation, USA) and GraphPad Prism version 5.03 or version 6.07 for Windows (GraphPad Software, La Jolla, CA, USA).

### 4.6. Synthesis of N^ε^-Acryloyllysine Piperazides ***2*** and ***3***

#### 4.6.1. General

All commercial reagents and solvents were used without further purification unless specified otherwise. Nuclear magnetic resonance spectra were recorded on an Agilent Technologies 400 MR spectrometer consisting of 400/54 premium compact magnet, 400 MR console, and 400 MHz OneNMRProbe PT probe head (400 MHz for ^1^H, 101 MHz for ^13^C and 376 MHz for ^19^F). Spectra were processed by using the program MestreNova (version 14.2.1-27684). NMR chemical shifts were referenced to the residual solvent resonances relative to tetramethylsilane (TMS; ^1^H and ^13^C) and trichlorofluoromethane (CFCl_3_; ^19^F). Mass spectra (ESI, positive mode) were obtained on a Waters Xevo TQ-S mass spectrometer driven by the Mass Lynx software. Thin-layer chromatography (TLC) was performed on Merck silica gel F-254 aluminum plates with visualization under UV (254 nm). Preparative column chromatography for compounds **2** and **3** was carried out on the Flash Chromatography “Selekt System” from Biotage using Biotage^®^ Sfär columns and solvent mixtures (specified below). The synthetic procedures and analytical data for compound **1** were described previously [64].

#### 4.6.2. Synthesis of *N*^α^-(2-Iodophenylacetyl)-*N*^ε^-acryloyl-l-lysin-4-(2-nitropyridin-5-yl)piperazide (**2**)

2-Iodophenylacetic acid (67 mg, 0.26 mmol, 1.2 eq.) was added to a solution of *N*^ε^-acryloyl-l-lysine-4-(2-nitropyridin-5-yl)piperazide (82 mg, 0.21 mmol, 1.0 eq.) [64] in DMF (3 mL). Subsequently, *N*,*N*-diisopropylethylamine (DIPEA, 89 µL, 0.51 mmol, 2.4 eq.) and *O*-(7-azabenzotriazol-1-yl)-*N*,*N*,*N*′,*N*′-tetramethyluronium hexafluorophosphate (HATU, 117 mg, 0.31 mmol, 1.5 eq.) were added, and the reaction mixture was stirred for 1 h. Afterward, DMF was removed in vacuo, and the residue was dissolved in ethyl acetate (20 mL). The organic phase was washed with saturated NaHCO_3_ (10 mL) and brine (10 mL), dried over Na_2_SO_4_, and evaporated. The crude product was purified via column chromatography (gradient from methanol-CH_2_Cl_2_ 1.2:98.8 to 10:90). The product-containing fractions were combined and evaporated to afford **2** (106 mg, 80%) as a yellow solid. *R*_f_ = 0.14 (methanol-CH_2_Cl_2_ 5:95); **^1^H NMR** (400 MHz, CDCl_3_) δ = 8.19 (d, ^3^*J* = 9.1 Hz, 1H, H-3 of pyridine), 8.14 (d, ^4^*J* = 3.0 Hz, 1H, H-6 of pyridine), 7.85 (d, ^3^*J* = 8.2 Hz, 1H, H-3 of 2-iodophenyl), 7.41–7.30 (m, 2H, 2×CH of 2-iodophenyl), 7.23 (dd, ^3^*J* = 9.1 Hz, ^4^*J* = 3.1 Hz, 1H, H-4 of pyridine), 7.04–6.95 (m, 1H, CH of 2-iodophenyl), 6.44 (d, ^3^*J* = 7.8 Hz, 1H, N_α_H), 6.24 (dd, ^3^*J* = 16.9 Hz, ^2^*J* = 1.5 Hz, 1H, CH*H* of acryloyl), 6.04 (dd, ^3^*J* = 17.0, 10.3 Hz, 1H, CH of acryloyl), 5.93 (s, 1H, N_ε_H), 5.60 (dd, ^3^*J* = 10.3 Hz, ^2^*J* = 1.5 Hz, 1H, C*H*H of acryloyl), 4.95 (td, ^3^*J* = 8.4, 4.4 Hz, 1H, C_α_H), 3.97–3.80 (m, 2H, CH_2_ of piperazine), 3.78–3.64 (m, 4H, CH_2_ of piperazine, CH_2_-phenyl), 3.60–3.23 (m, 6H, 2×CH_2_ of piperazine, C_ε_H_2_), 1.86–1.55 (m, 4H, C_β_H_2_, C_δ_H_2_), 1.42–1.34 (m, 2H, C_γ_H_2_); **^13^C NMR** (101 MHz, CDCl_3_) δ = 170.40 (CO), 169.80 (CO), 166.04 (CO), 149.56 (quart. C of pyridine), 148.64 (quart. C of pyridine), 140.00 (C-3 of 2-iodophenyl), 137.95 (C-1 of 2-iodophenyl), 134.28 (C-6 of pyridine), 131.15 (CH of 2-iodophenyl), 130.80 (CH of acryloyl), 129.54 (CH of 2-iodophenyl), 129.12 (CH of 2-iodophenyl), 126.75 (CH_2_ of acryloyl), 121.48 (C-4 of pyridine), 119.83 (C-3 of pyridine), 101.20 (C-2 of 2-iodphenyl), 48.55 (C_α_), 48.51 (CH_2_-phenyl), 46.98 (CH_2_ of piperazine), 46.64 (CH_2_ of piperazine), 44.73 (CH_2_ of piperazine), 41.47 (CH_2_ of piperazine), 39.12 (C_ε_), 32.67 (C_β_), 28.64 (C_δ_), 22.39 (C_γ_); **MS (ESI^+^)**: *m*/*z* calculated for C_26_H_32_IN_6_O_5_ = 635.14 [M+H]^+^; found 635.1 [M+H]^+^.

#### 4.6.3. Synthesis of *N*^α^-(2-Iodophenylacetyl)-*N*^ε^-acryloyl-l-lysin-4-(2-trifluoromethyl-5-yl)piperazide (**3**)

2-Iodophenylacetic acid (54 mg, 0.21 mmol, 1.0 eq.) was added to a solution of *N*^ε^-acryloyl-l-lysin-4-(2-trifluoromethyl-5-yl)piperazide (85 mg, 0.21 mmol, 1 eq.) [64] in DMF (3 mL). Subsequently, DIPEA (72 µL, 0.41 mmol, 2.0 eq.) and (benzotriazole-1-yloxy)tripyrrolidinophosphonium hexafluorophosphate (PyBOP, 128 mg, 0.25 mmol, 1.2 eq.) were added, and the reaction mixture was stirred for 1 h. Afterward, DMF was removed in vacuo, and the residue was dissolved in ethyl acetate (20 mL). The organic phase was washed with saturated NaHCO_3_ (10 mL) and brine (10 mL), dried over Na_2_SO_4_, and evaporated. The crude product was purified via column chromatography (gradient from acetone-ethyl acetate 0:100 to 52:48). The product-containing fractions were combined and evaporated to afford **3** (94 mg, 68%) as a white crystalline solid. *R*_f_ = 0.60 (acetone-ethyl acetate 50:50); **^1^H NMR** (400 MHz, DMSO-*d*_6_) δ = 8.43 (d, ^4^*J* = 2.9 Hz, 1H, H-6 of pyridine), 8.35 (d, ^3^*J* = 8.2 Hz, 1H, N_α_H), 8.04 (t, ^3^*J* = 5.9 Hz, 1H, N_ε_H), 7.81 (dd, ^3^*J* = 7.9 Hz, ^4^*J* = 1.2 Hz, 1H, H-3 of 2-iodophenyl), 7.66 (d, ^3^*J* = 8.8 Hz, 1H, H-3 of pyridine), 7.43 (dd, ^3^*J* = 9.0 Hz, ^4^*J* = 2.9 Hz, 1H, H-4 of pyridine), 7.36–7.28 (m, 2H, 2×CH of 2-iodophenyl), 7.03–6.93 (m, 1H, CH of 2-iodophenyl), 6.18 (dd, ^3^*J* = 17.1, 10.1 Hz, 1H, CH of acryloyl), 6.03 (dd, ^3^*J* = 17.1 Hz, ^2^*J* = 2.3 Hz, 1H, CH*H* of acryloyl), 5.53 (dd, ^3^*J* = 10.1 Hz, ^2^*J* = 2.3 Hz, 1H, C*H*H of acryloyl), 4.80–4.70 (m, 1H, C_α_H), 3.79–3.52 (m, 6H, 2×CH_2_ of piperazine, CH_2_-phenyl), 3.48–3.26 (m, 4H, 2×CH_2_ of piperazine), 3.17–3.04 (m, 2H, C_ε_H_2_), 1.74–1.22 (m, 6H, C_β_H_2_, C_γ_H_2_, C_δ_H_2_); **^13^C NMR** (101 MHz, DMSO-*d*_6_) δ = 169.87 (CO), 168.54 (CO), 164.41 (CO), 147.98 (C-1 of 2-iodophenyl), 139.37 (C-5 of pyridine), 138.80 (C-3 of 2-iodophenyl), 136.90 (C-6 of pyridine), 135.31 (d, ^2^*J*_C,F_ = 34.0 Hz, C-2 of pyridine), 131.86 (CH of acryloyl), 130.86 (CH of 2-iodophenyl), 128.52 (CH of 2-iodophenyl), 128.15 (CH of 2-iodophenyl), 124.76 (CH_2_ of acryloyl), 122.37 (d, ^1^*J*_C,F_ = 272.4 Hz, CF_3_), 120.94 (d, ^3^*J*_C,F_ = 2.9 Hz, C-3 of pyridine), 120.76 (C-4 of pyridine), 101.55 (C-2 of 2-iodophenyl), 48.16 (C_α_), 46.63 (CH_2_ of piperazine), 46.42 (CH_2_-phenyl), 46.19 (CH_2_ of piperazine), 44.21 (CH_2_ of piperazine), 40.87 (CH_2_ of piperazine), 38.33 (C_ε_), 31.31 (C_β_), 28.85 (C_δ_), 22.66 (C_γ_); **^19^F NMR** (376 MHz, DMSO-*d*_6_) δ = -64.88 (s, CF_3_); **MS (ESI^+^):** *m*/*z* calculated for C_27_H_32_F_3_IN_5_O_3_ = 658.15 [M+H]^+^; found 658.1 [M+H]^+^.

### 4.7. Statistical Analysis

Prism version 9.4.1 was utilized to perform the two-way repeated measures ANOVA tests. Sphericity was not assumed. Alpha threshold was set to 0.05.

## Figures and Tables

**Table 1:** Rate constants for binding of GTPγS to hTGase 2.

Labeling Strategy	*k_on_* (10^6^ M^−1^ s^−1^)	*k_off_* (10^−2^ s^−1^)	*K*_d_ (nM)
non-directed	17 ± 4	1.6 ± 0.6	1.0 ± 0.4
His-tag-directed	16 ± 2	1.9 ± 0.5	1.2 ± 0.4

*k_on_* and *k_off_* are the best-fit parameters (±standard errors) of one association and dissociation experiment (data shown in Figure 3; for calculation, see Supplementary Materials Equations (S12) to (S21)). Staffler et al. [57] reported values of *k_on_* = 41 × 10^6^ M^−1^s^−1^, *k_off_* = 5.1 × 10^−2^ s^−1^, and *K*_d_ = 1.2 nM.

**Table 2:** Summary of inhibition parameters of compounds **1**–**3** toward commercial and self-produced hTGase 2.

	IC_50_ (nM) ^a,c^	IC_50_ (nM) ^b,c^	*k*_inact_/*K*_I_ (M^−1^s^−1^) ^a,d^
**1**	108 (3) ^e^	135 (8)	3850 (240) ^e^
**2** (NO_2_)	154 (11)	116 (4)	8340 (1160)
**3** (CF_3_)	280 (24)	209 (13)	4500 (0)

^a^ Commercial His_6_-hTGase 2. ^b^ In-house produced *N*-terminally Twin-Strep-tagged hTGase 2. ^c^ Determined with a FA-based assay [66]. Data shown are mean values (±standard error of the mean) of three separate experiments, each performed in duplicate; the pre-incubation time of the enzyme and the inhibitor was 5 min. ^d^ Determined with a fluorimetric assay [65]. Data shown are mean values (±standard error of the mean) of two to four separate experiments, each performed in duplicate. ^e^ Data have been previously reported [64].

## Data Availability

Not applicable.

## Supplementary Materials

The following supporting information can be downloaded at: https://www.mdpi.com/article/10.3390/ijms24021650/s1.
