# Supplementary material for: The Impact of Nε-Acryloyllysine Piperazides on the Conformational Dynamics of Transglutaminase 2
Source: Int J Mol Sci. 2023 Jan 13;24(2):1650. doi: 10.3390/ijms24021650 (PMC9865645; doi:10.3390/ijms24021650)
Supplement: Supplementary file 1 [file ijms-24-01650-s001.zip › ijms-2141786-supplementary.pdf]

Supplementary Materials

The impact of N^{ε}-acryloyllysine piperazides on the conformational dynamics of transglutaminase 2

Andreas Heerwig [1,#], Alfred Kick [1], Paul Sommerfeld [2,#], Sophia Eimermacher [2], Frederick Hartung [2], Markus Laube [3,4], Dietmar Fischer [2], Hans-Jürgen Pietzsch [3,4], Jens Pietzsch [3,4], Reik Löser [3,4], Michael Mertig [1,4], Markus Pietsch [2,*] and Robert Wodtke [3,*]

1 *Kurt-Schwabe-Institut für Mess- und Sensortechnik Meinsberg e.V., 04736 Waldheim, Germany*

2 *Institutes I & II of Pharmacology, Center of Pharmacology, Faculty of Medicine and University Hospital of Cologne, University of Cologne, 50931 Cologne, Germany*

3 *Helmholtz-Zentrum Dresden-Rossendorf, Institute of Radiopharmaceutical Cancer Research, Bautzner Landstraße 400, 01328 Dresden, Germany*

4 *School of Science, Faculty of Chemistry and Food Chemistry, Technische Universität Dresden, 01062 Dresden, Germany*

# *These authors contributed equally to this work.*

* *Correspondence: markus.pietsch@uk-koeln.de (M.P.); r.wodtke@hzdr.de (R.W.);*

Tel.: +49-221-478-97737 (M.P.); +49-351-260-4033 (R.W.)

Table of Contents

Analysis of association kinetics by fluorescence proximity sensing......................3

Analysis of the dissociation kinetics by fluorescence proximity sensing 5

Damped Gauss-Newton algorithm and error estimation..7

The double logistic function, its derivatives and Jacobian matrix...........................9

Analysis of dose-response curves 11

Discussion S1: Conditions for the *native (GTP-)PAGE* experiments 12

Figure S1: Purification of hTGase 2-DNA conjugates.................................13

Figure S2: Exemplary time-dependent fluorescence traces for the upward movement of dsDNA and hTGase 2-dsDNA nanolevers..........14

Figure S3. Inhibition of hTGase 2 by compounds 1-3. 15

Figure S4. Statistical analyses for the switchSENSE® experiments with inhibitors 1 and 2 16

Figure S5. Influence of the inhibitors 1-3 on the activity and conformation of hTGase 2 at different temperatures. 17

Figure S6. Influence of inhibitors 1-3 on the activity and conformation of hTGase 2 in the absence and presence of Ca^{2+} or/and GTPγS.18

Figure S7. Effect of increasing concentrations of inhibitor 2 on the activity and the conformation of hTGase 2...20

Figure S8. Calculation of the net charge of in-house produced *N*-terminally Twin-Strep-tagged hTGase 2 at a pH of 8.3...............................22

References 23

Analysis of association kinetics by fluorescence proximity sensing

Based on the reaction equation expressed by Equation (1), one can set up the kinetic differential equation shown in Equation (S1).

$$\frac{d[PL]}{dt} = \frac{d\xi}{dt} = [P][L]k_{on} - [PL]k_{off}$$
$$= ([P]_0 - \xi)([L]_0 - \xi)k_{on} - ([PL]_0 + \xi)k_{off} \quad \text{(S1)}$$

In Equation (S1), $[P] = [P]_0 - \xi$, $[L] = [L]_0 - \xi$, and $[PL] = [PL]_0 + \xi$ represent the concentrations of the free protein, the ligand, and of the protein-ligand complex, respectively. $[P]_0$, $[L]_0$, and $[PL]_0$ denote their concentrations at the beginning of the association at time $t = 0$, and ξ is the extent of the reaction with respect to Equation (1).

Association:

During the association experiment the concentration of the ligand concentration is assumed to be constant, $[L] = c_{GTP\gamma S}$. Therefore, Equation (S1) simplifies to Equation (S2).

$$\frac{d\xi}{dt} = ([P]_0 - \xi)\, c_{GTP\gamma S}\, k_{on} - \xi \cdot k_{off} = [P]_0\, c_{GTP\gamma S}\, k_{on} - \xi\left(c_{GTP\gamma S}\, k_{on} + k_{off}\right) \quad \text{(S2)}$$

Separation of variables is used to solve Equation (S2) by integration of Equation (S3):

$$\int_0^{[PL]_a} \frac{d\xi}{[P]_0\, c_{GTP\gamma S}\, k_{on} - \xi\left(c_{GTP\gamma S}\, k_{on} + k_{off}\right)} = \int_0^{t_a} dt \quad \text{(S3)}$$

This leads to the time dependent protein-ligand complex concentration in Equation (S4).

$$[PL]_a = \frac{[P]_0\, c_{GTP\gamma S}\, k_{on}}{c_{GTP\gamma S}\, k_{on} + k_{off}}\left[1 - e^{-(c_{GTP\gamma S}\, k_{on} + k_{off})t_a}\right] \quad \text{(S4)}$$

Approaching the equilibrium, Equation (S4) gives the equilibrium concentration of the protein-ligand complex expressed by Equation (S5).

$$\lim_{t_a \to \infty}([PL]_a) = [PL]_\infty = \frac{[P]_0\, c_{GTP\gamma S}\, k_{on}}{c_{GTP\gamma S}\, k_{on} + k_{off}} \quad \text{(S5)}$$

Finally, the substitution of Equation (S5) into Equation (S4) results in Equation (2). The Jacobian matrix of Equation (5) is given by Equation (S6).

$$J_{Fa} = \left[\frac{\partial F_a}{\partial F_{a,e}} \; \frac{\partial F_a}{\partial F_{a,s}} \; \frac{\partial F_a}{\partial k_a}\right] \tag{S6}$$

The corresponding partial derivatives are given in Equations (S6a), (S6b), and (S6c).

$$\frac{\partial F_a}{\partial F_{a,e}} = 1 - e^{-k_a \cdot t_a} \tag{S6a}$$

$$\frac{\partial F_a}{\partial F_{a,s}} = e^{-k_a \cdot t_a} \tag{S6b}$$

$$\frac{\partial F_a}{\partial k_a} = t\left(F_{d,e} - F_{d,s}\right)\left(e^{-k_a \cdot t_a}\right) \tag{S6c}$$

The fitting parameters of Equation (5) are summarized in a vector as shown in Equation (S6d).

$$b_{Fa} = (F_{a,e} \; F_{a,s} \; k_a)^T \tag{S6d}$$

Analysis of the dissociation kinetics by fluorescence proximity sensing

The ligand concentration in the dissociation is assumed to be zero. Therefore, the reaction equation expressed by Equation (1) is reduced to only the dissociation reaction. Consequently, the differential Equation (S1) simplifies to Equation (S7).

$$\frac{d[PL]}{dt} = -[PL]k_{off} \tag{S7}$$

Again, separation of variables is possible to solve Equation (S7) by integration in Equation (S8) leading to Equation (S9), were $[PL]_{d,0} \neq 0$.

$$\int_{[PL]_{0,d}}^{[PL]_d} \frac{d[PL]}{-[PL] \cdot k_{off}} = \int_0^{t_d} dt \tag{S8}$$

$$ln\left(\frac{[PL]_d}{[PL]_{d,0}}\right) = -k_{off} \cdot t_d \tag{S9}$$

Finally, Equation (S9) gives the dissociation time (t_d)-dependent protein-ligand complex concentration in Equation (S10), which results in Equation (6).

$$[PL]_d = [PL]_{d,0}e^{-k_{off}\,t_d} \tag{S10}$$

The Jacobian matrix of Equation (8) is given by Equation (S11).

$$J_{Fd} = \left[\frac{\partial F_d}{\partial F_{d,e}}\ \frac{\partial F_d}{\partial F_{d,s}}\ \frac{\partial F_d}{\partial k_{off}}\right] \tag{S11}$$

The corresponding partial derivatives with respect to the vector are given in Equations (S11a), (S11b), and (S11c).

$$\frac{\partial F_d}{\partial F_{d,e}} = 1 - e^{-k_{off} \cdot t_d} \tag{S11a}$$

$$\frac{\partial F_d}{\partial F_{d,s}} = e^{-k_{off} \cdot t_d} \tag{S11b}$$

$$\frac{\partial F_d}{\partial k_{off}} = t\left(F_{d,e} - F_{d,s}\right)\left(e^{-k_{off} \cdot t_d}\right) \tag{S11c}$$

The fitting parameters of Equation (8) are summarized in a vector as shown in Equation (S11d).

$$b_{Fd} = (F_{d,e}\ F_{d,s}\ k_{off})^T \tag{S11d}$$

Damped Gauss-Newton algorithm and error estimation

The fitting procedure starts with choosing reasonable initial values for these parameters. Then the fitting is iteratively realized by a damped Gauss-Newton algorithm to find the best parameters in terms of the non-linear least squares fit based on Equation (S12).

$$b_{n+1} = b_n + d_n \cdot \left(J_n{}^T J_n\right)^{-1} \cdot J_n{}^T (y_m - y_n) \qquad \text{(S12)}$$

In Equation (S12), n is the iteration step number starting with n = 0 as the initially chosen parameter values. Thus, b_n is the vector of the fitting parameters, d_n $(0 < d_n \leq 1)$ is a damping factor to ensure minimization of the squared deviations of the measured values y_m from the predicted values $y_n = y(x, b_n)$ by a model function equation. J_n is the respective Jacobian matrix of the first derivatives with respect to b_n. Furthermore, the diagonal elements of the variance-covariance matrix are calculated for a locally linear range, and the standard errors of the parameters (s_b) are determined by Equation (S13) [1, 2].

$$s_b = \sqrt{\frac{\sum_{j=1}^{N}\left(y_{m,j} - y(x_j)\right)^2}{\nu} diag\left(\left(J_n{}^T J_n\right)^{-1}\right)} \qquad \text{(S13)}$$

In Equation (S13), $\nu = \mathrm{N} - \mathrm{N_b}$ is the degree of freedom, where N is the number of evaluated data points, and N_b is the number of parameters to fit.

According to Equation (3), the association rate constant is calculated by Equation (S14).

$$k_{on} = \frac{k_a - k_{off}}{c_{GTP\gamma S}} \qquad \text{(S14)}$$

The corresponding standard error, s_{kon}, can be estimated by the simplified propagation of uncertainty by Equation (S15).

$$s_{kon} = \frac{\sqrt{s_{ka}{}^2 + s_{koff}{}^2}}{c_{GTP\gamma S}}, \qquad \text{(S15)}$$

In Equation (S15), s_{ka} and s_{koff} are the estimated standard errors of the apparent rate constant in the association experiment and the rate constants of the dissociation reactions, respectively.

It is possible to calculate the dissociation constant, K_d, by Equation (S16) with determined rate constants.

$$K_d = \frac{k_{off}}{k_{on}} \tag{S16}$$

Using Equation (S14) in Equation (S16) one gets Equation (S17).

$$K_d = \frac{c_{GTP\gamma S} k_{off}}{k_a - k_{off}} \tag{S17}$$

Again, the corresponding standard error, s_{Kd}, can be estimated by the simplified propagation of uncertainty expressed by Equation (S18).

$$s_{Kd}{}^2 = \left(\frac{\partial K_d}{\partial k_a}\right)^2 s_{ka}{}^2 + \left(\frac{\partial K_d}{\partial k_{off}}\right)^2 s_{koff}{}^2 \tag{S18}$$

The corresponding derivatives are given in Equations (S19) and (S20).

$$\frac{\partial K_d}{\partial k_{off}} = \frac{c_{GTP\gamma S} k_a}{\left(k_a - k_{off}\right)^2} \tag{S19}$$

$$\frac{\partial K_d}{\partial k_a} = -\frac{c_{GTP\gamma S} k_{off}}{\left(k_a - k_{off}\right)^2} \tag{S20}$$

Subsequent substitutions result in Equation (S21) for the estimation of the standard error of the dissociation constant.

$$s_{Kd} = K_d \sqrt{\left(\frac{s_{ka}}{k_a - k_{off}}\right)^2 + \left(\frac{k_a s_{koff}}{k_{off}\left(k_a - k_{off}\right)}\right)^2} \tag{S21}$$

The double logistic function, its derivatives and Jacobian matrix

The substitution $e^{\frac{x-x_1}{k_1}} = E_1$ and $e^{\frac{x-x_2}{k_2}} = E_2$ in Equation (9) gives Equation (S22).

$$y = y_0 + A\left[\frac{p}{1+E_1} + \frac{1-p}{1+E_2}\right] \tag{S22}$$

Then, the first, second, and third derivatives with respect to x are given in Equations (S23), (S24), and (S25), respectively.

$$\frac{dy}{dx} = -A\left[\frac{p}{k_1}\frac{E_1}{(1+E_1)^2} + \frac{(1-p)}{k_2}\frac{E_2}{(1+E_2)^2}\right] \tag{S23}$$

$$\frac{d^2y}{dx^2} = -A\left[\frac{p}{{k_1}^2}\frac{E_1(1-E_1)}{(1+E_1)^3} + \frac{(1-p)}{{k_2}^2}\frac{E_2(1-E_2)}{(1+E_2)^3}\right] \tag{S24}$$

$$\frac{d^3y}{dx^3} = -A\left[\frac{p}{{k_1}^3}\frac{(1-2E_1)(1+E_1)-3E_1(1-E_1)}{(1+E_1)^4} + \frac{(1-p)}{{k_2}^3}\frac{(1-2E_2)(1+E_2)-3E_2(1-E_2)}{(1+E_2)^4}\right] \tag{S25}$$

Equations (S23) to (S25) are used to determine the inflection point in Equation (S22), where the absolute value of the first derivative is at its maximum. Therefore, the slopes are calculated by Equation (S23) at all measured x, and their maximum absolute value is chosen as the starting value, $x_{in,0}$, to numerically find the corresponding inflection point by the classical Newton-Raphson method as shown in Equation (S26).

$$x_{in,r+1} = x_{in,r} - \frac{\frac{d^2y}{dx^2}\left(x_{in,r}\right)}{\frac{d^3y}{dx^3}\left(x_{in,r}\right)} \tag{S26}$$

In Equation (S26), $x_{in,r+1}$ is the iteratively calculated time value of the inflection point with the previous iteration step number r. For the investigated data, the above described procedure to choose the starting value $x_{in,0}$ provides a sufficiently close value, so that usually after r = 6 iterations the machine precision of 64-bit computing is reached. The Jacobian matrix of Equation (9) or (S22) is given by Equation (S27).

$$J_y = \left[\frac{\partial y}{\partial y_0}\ \frac{\partial y}{\partial A}\ \frac{\partial y}{\partial p}\ \frac{\partial y}{\partial x_1}\ \frac{\partial y}{\partial x_2}\ \frac{\partial y}{\partial k_1}\ \frac{\partial y}{\partial k_2}\right] \tag{S27}$$

The corresponding partial derivatives are given in Equations (S27a) to (S27g).

$$\frac{\partial y}{\partial y_0} = \begin{pmatrix} 1 \\ \vdots \\ 1 \end{pmatrix}, \text{ an all-ones vector} \tag{S27a}$$

$$\frac{\partial y}{\partial A} = \frac{p}{1 + E_1} + \frac{1 - p}{1 + E_2} \tag{S27b}$$

$$\frac{\partial y}{\partial p} = A \left[\frac{1}{1 + E_1} - \frac{1}{1 + E_2} \right] \tag{S27c}$$

$$\frac{\partial y}{\partial x_1} = A \frac{p}{k_1} \frac{E_1}{(1 + E_1)^2} \tag{S27d}$$

$$\frac{\partial y}{\partial x_2} = A \frac{1 - p}{k_2} \frac{E_2}{(1 + E_2)^2} \tag{S27e}$$

$$\frac{\partial y}{\partial k_1} = A \frac{p}{{k_1}^2} \frac{(x - x_1) E_1}{(1 + E_1)^2} \tag{S27f}$$

$$\frac{\partial y}{\partial k_2} = A \frac{1 - p}{{k_2}^2} \frac{(x - x_2) E_2}{(1 + E_2)^2} \tag{S27g}$$

The fitting parameters of Equation (9) or (S22) are summarized in a vector as shown in Equation (S28).

$$b_y = (y_0 \; A \; p \; x_1 \; x_2 \; k_1 \; k_2)^T \tag{S28}$$

Analysis of dose-response curves

$$y = \frac{(Top\ - Bottom)}{1 + \frac{{IC_{50}}^n}{[I]^n}} + Bottom \tag{S29}$$

In Equation S29, Top and Bottom represent the upper and the lower plateau of the dose-response curve, i.e., the value of y in the absence and the presence of an indefinitely high inhibitor concentration, [I], respectively. The inhibitor concentrations resulting in half-maximum inhibition, IC_{50}, for curves shown in Figures 8D, 8F, and S3 were calculated considering the curve's steepness at the point of inflection, i.e., the Hill slope, n.

Discussion S1: Conditions for the *native (GTP-)PAGE* experiments

All *native (GTP-)PAGE* experiments were accompanied by an investigation of the respective samples via SDS-PAGE to unequivocally identify covalently modified hTGase 2 species by intra- and/or inter-crosslinking [3]. As the incubation temperature of the samples affects the band pattern found in both SDS- and *native GTP-PAGE*, samples of self-produced hTGase 2 were incubated at 20 °C and 37 °C in an initial experiment [3] (see Figure S4). In the absence of Ca^{2+} (lanes 1, 2) and the presence of both Ca^{2+} and compounds **1-3** (lanes 4-9), the higher incubation temperature led to the formation of a larger amount of primarily noncovalent multimers of hTGase 2 appearing as multiple bands with low electrophoretic mobility in the *native GTP-PAGE* but are hardly visible in the SDS-PAGE. Separation of hTGase 2 species by SDS-PAGE, incubated in the presence of Ca^{2+} without any inhibitor, revealed inter-crosslinked hTGase 2 already at 20 °C (lane 3). Such covalent multimerization was highly increased at elevated incubation temperature (data not shown), as reported before [3]. As a result of this initial experiment, all further investigation was done at 20 °C to slow down protein multimerization.

Figure S1: Purification of hTGase 2-DNA conjugates

Typical purification results with proFIRE after DNA-hTGase 2 coupling with amine coupling kit 1 (CK-NH2-1-B48; top) or amine coupling kit 2 for His-tagged proteins (PF-NH2-2-B48; bottom).

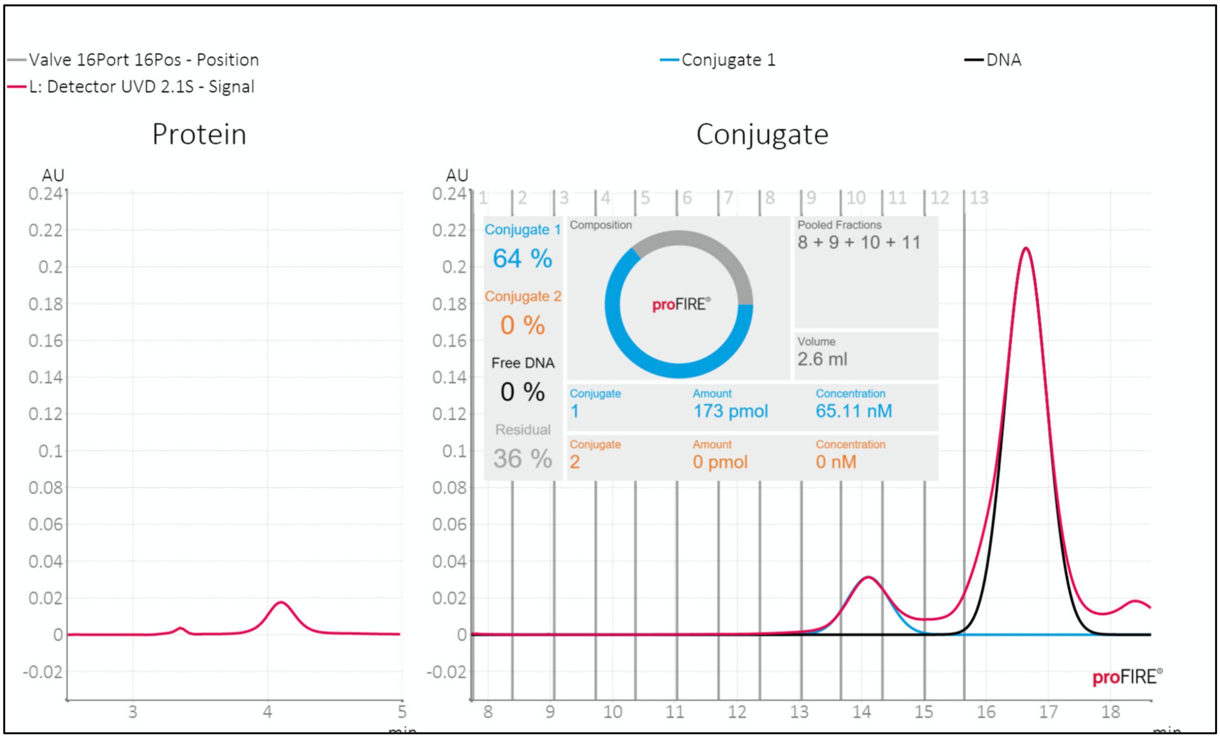

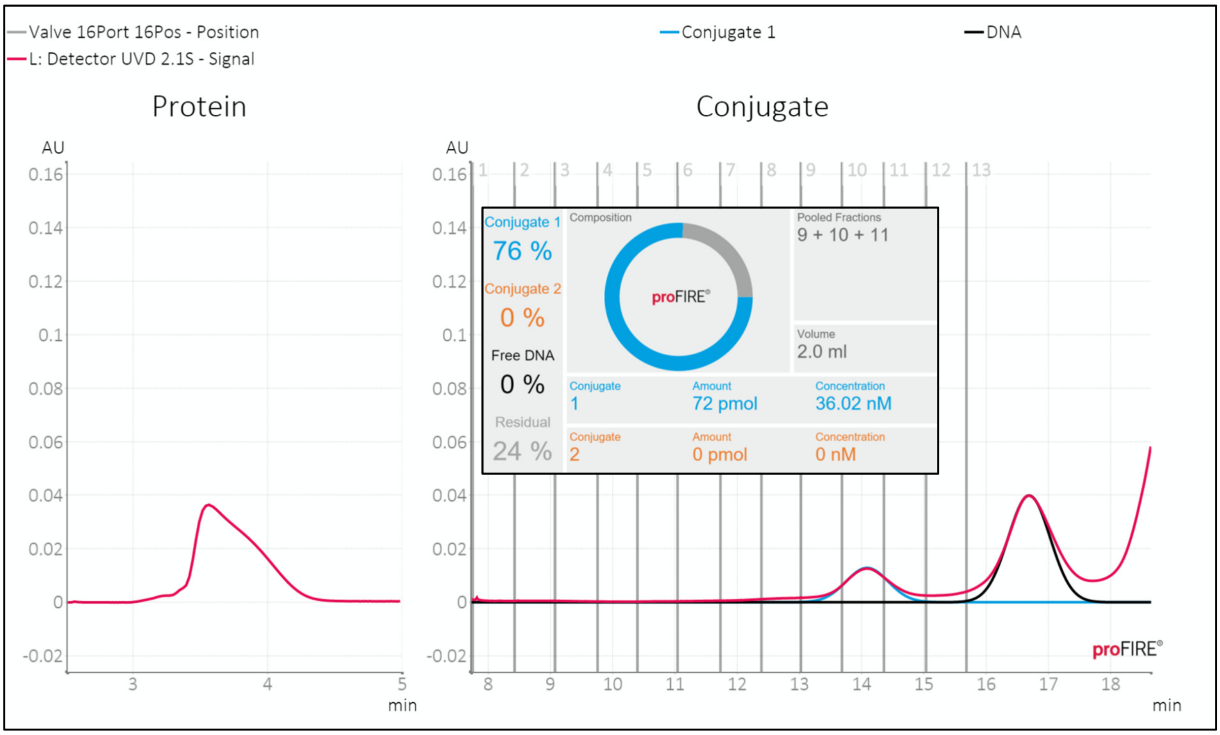

Figure S2: Exemplary time-dependent fluorescence traces for the upward movement of dsDNA and hTGase 2-dsDNA nanolevers

Exemplary time-dependent normalized fluorescence traces of the bare dsDNA nanolever and the hTGase 2-dsDNA nanolever (non-directed labeling), as well as the hTGase 2-dsDNA nanolever in the presence of GTPγS (20 nM) or Ca^{2+} (1 mM) in TE40 buffer. Curves with the best-fit parameters (referred to as "fit") of a double logistic function are shown.

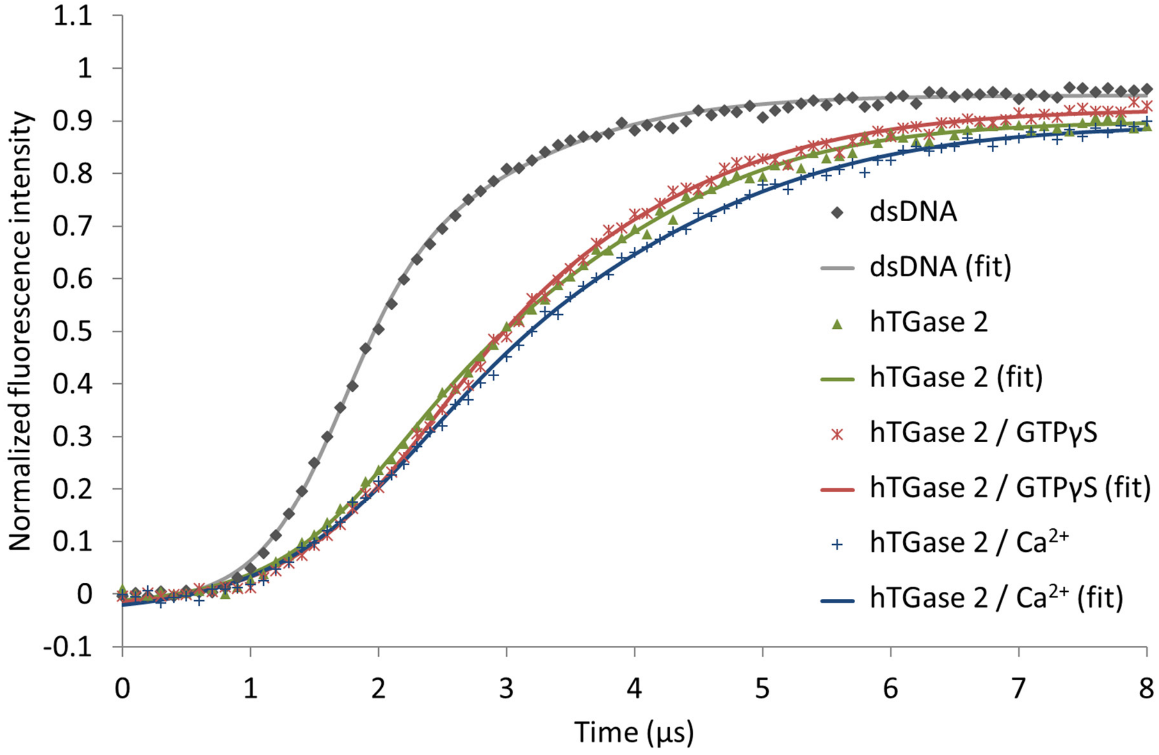

Figure S3. Inhibition of hTGase 2 by compounds 1-3.

The hTGase 2 and the respective inhibitor were pre-incubated for 5 min (**A**, using in-house produced protein) or 30 min (**B**, using in-house produced and commercial protein, respectively) before starting the enzymatic reaction [4]. Data shown are mean values ± standard error of the mean of three separate experiments, each performed in duplicate. IC_{50} values were calculated according to Equation (S29).

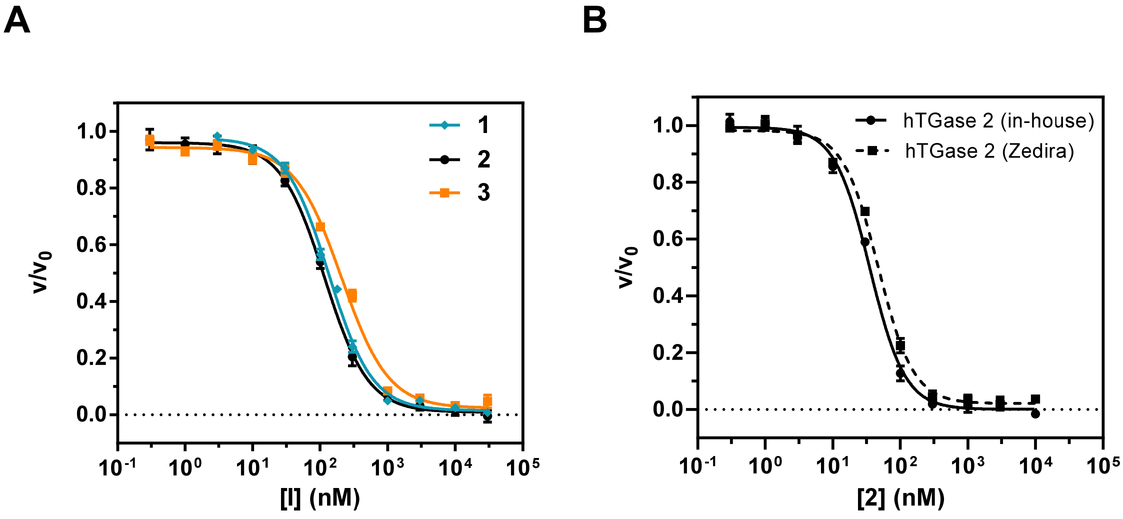

	IC_{50} (nM)[a]	IC_{50}/[hTGase 2][a]
hTGase 2 (in-house)[b]	35.2 (1.9)	0.571 (0.031)
hTGase 2 (Zedira)[c]	47.4 (3.4)	0.740 (0.054)

[a]Mean values (± standard error of the mean) of three separate experiments, each performed in duplicate for experiments shown in **B**. [b,c]The concentration of hTGase 2 was 61.6 nM[b] and 64.1 nM[c], respectively. The fraction of active hTGase 2 was assumed to be one for each of the two proteins.

Figure S4. Statistical analyses for the switchSENSE® experiments with inhibitors 1 and 2

For statistical comparison of the relative inverse slopes, the values were subjected to a two-way repeated measures ANOVA with Tukey's multiple comparison test (matched values for the relative inverse slopes obtained for the same electrode). P values are given above the brackets and values <0.05 were considered statistically significant.

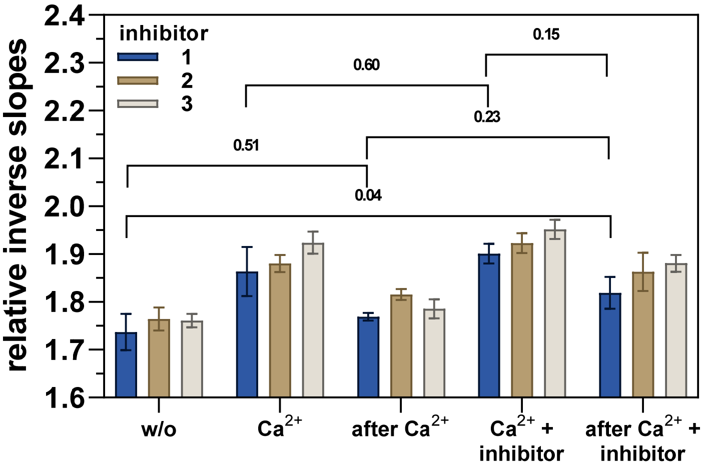

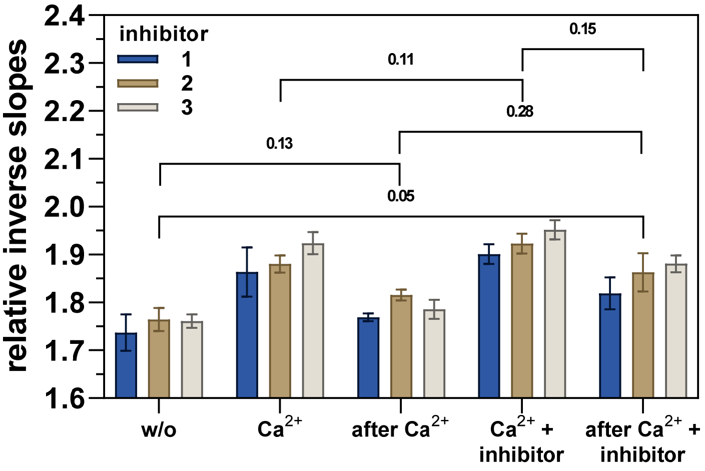

Figure S5. Influence of the inhibitors 1-3 on the activity and conformation of hTGase 2 at different temperatures.

A) SDS-PAGE was done on 7% separating gel, with both the gel and the running buffer containing neither GTP nor $MgCl_2$. **B**) *Native GTP-PAGE* was done on 7% separating gel, with both the gel and the running buffer containing 50 µM GTP and 500 µM $MgCl_2$. For both the SDS-PAGE and the *native GTP-PAGE*, samples of 8.23 µM self-produced hTGase 2 were first incubated for 30 min at 20 or 37 °C in 100 mM MOPS pH 8.0, 100 mM NaCl, 5.33% (v/v) glycerol with $CaCl_2$ and inhibitors **1**-**3** (2.12 % DMSO) as indicated. GTP and $MgCl_2$ were then added to all samples, which were then again incubated for 60 min at 20 or 37 °C. **A**) After addition of SDS loading buffer and denaturation for 5 min at 60 °C, a volume of 5 µL of each sample containing 2.5 µg of hTGase 2 was loaded onto the gel. **B**) For the *native GTP-PAGE*, native loading buffer was added, and a volume of 10 µL of each sample containing 5 µg of hTGase 2 was loaded onto the gel. After the electrophoretic separation, gels were stained with Coomassie Brilliant Blue G250 and scanned. M, 3 µL of PageRuler™ Plus Prestained Protein Ladder (neither treated with SDS loading buffer nor denaturated); SF, solvent front.

**A** SDS-PAGE											**B** native GTP-PAGE								
1	2	3	4	5	6	7	8	9	M	lane	1	2	3	4	5	6	7	8	9
		x	x	x	x	x	x	x		3 mM Ca^{2+}			x	x	x	x	x	x	x
			x	x						84.8 µM inhibitor **1**				x	x				
					x	x				84.8 µM inhibitor **2**						x	x		
							x	x		84.8 µM inhibitor **3**								x	x
20	37	20	20	37	20	37	20	37		30 min incubation at °C	20	37	20	20	37	20	37	20	37
Addition of 500 µM GTP and 1 mM Mg^{2+} to each sample																			
20	37	20	20	37	20	37	20	37		60 min incubation at °C	20	37	20	20	37	20	37	20	37
then addition of SDS loading buffer and sample denaturation for 5 min at 60 °C (**A**) or loading buffer (**B**)																			

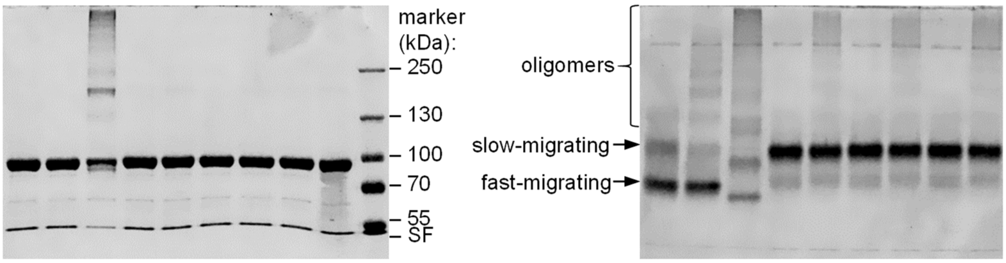

Figure S6. Influence of inhibitors 1-3 on the activity and conformation of hTGase 2 in the absence and presence of Ca^{2+} or/and GTPγS.

Images of *native (GTP-)PAGE*, with both the gel and the running buffer containing either no GTP (**A**) or 50 µM GTP (**B**), are the same as in Figures 7A and 7B, respectively, of the main text. The readout parameter for image quantification was the signal, which represents “*the sum of the individual pixel intensity values for a shape minus the product of the average intensity values of the pixels in the background and the total number of pixels enclosed by the shape*“ (Calculation Descriptions within the help of the Image Studio v.5.2.5 software). The magenta rectangle displays the area used as the background. The total signal of each lane is represented by the red rectangle covering the whole area of the lane. Shapes of “oligomers”, “slow-migrating”, “intermediate-migrating,” and “fast-migrating” species were manually added and adjusted in size and position. **C**) SDS-PAGE was done on 7% separating gel. Both the gel and the running buffer contained no GTP. Samples of 8.23 µM self-produced hTGase 2 were incubated for 30 min at 20 °C in 100 mM MOPS pH 8.0, 100 mM NaCl, 5.33% (v/v) glycerol with $CaCl_2$, DMSO or inhibitors **1-3** (2.12 % DMSO), and GTPγS as indicated. After addition of SDS loading buffer and denaturation for 5 min at 60 °C, a volume of 3 µL of each sample containing 1.5 µg of hTGase 2 was loaded onto the gel. After the electrophoretic separation, gels were stained with Coomassie Brilliant Blue G250 and scanned. M, 3 µL of PageRuler™ Plus Prestained Protein Ladder (neither treated with SDS loading buffer nor denaturated); SF, solvent front.

A native PAGE

1	2	3	4	5	6	7	8	9	10	11	12	13	14	lane
					x	x	x	x		x	x	x	x	3 mM Ca^{2+}
									x	x	x	x	x	500 µM GTPγS
	x				x				x	x				DMSO
		x				x					x			84.8 µM inhibitor **1**
			x				x					x		84.8 µM inhibitor **2**
				x				x					x	84.8 µM inhibitor **3**

30 min incubation at 20 °C then addition of loading buffer

B native GTP-PAGE

1	2	3	4	5	6	7	8	9	10	11	12	13	14	lane
					x	x	x	x		x	x	x	x	3 mM Ca^{2+}
									x	x	x	x	x	500 µM GTPγS
	x				x				x	x				DMSO
		x				x					x			84.8 µM inhibitor **1**
			x				x					x		84.8 µM inhibitor **2**
				x				x					x	84.8 µM inhibitor **3**

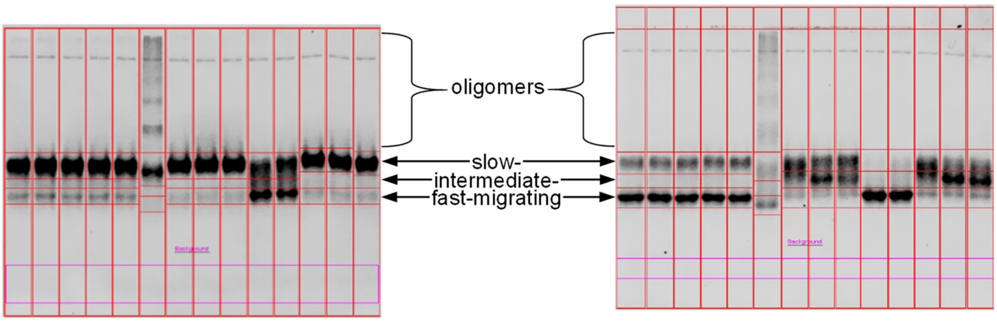

C SDS-PAGE

1	2	3	4	5	6	7	8	9	10	11	12	13	14	M	lane
					x	x	x	x		x	x	x	x		3 mM Ca^{2+}
									x	x	x	x	x		500 µM GTPγS
	x				x				x	x					DMSO
		x				x					x				84.8 µM inhibitor **1**
			x				x					x			84.8 µM inhibitor **2**
				x				x					x		84.8 µM inhibitor **3**

30 min incubation at 20 °C, then addition of SDS loading buffer and sample denaturation for 5 min at 60 °C

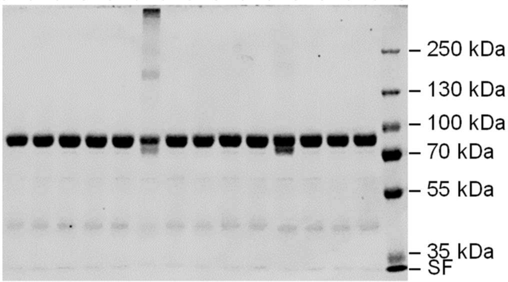

Figure S7. Effect of increasing concentrations of inhibitor 2 on the activity and the conformation of hTGase 2.

Images of *native GTP-PAGE*, with both the gel and the running buffer containing 50 µM GTP and either no $MgCl_2$ (**A**) or 500 µM $MgCl_2$ (**B**), are the same as in Figures 8A and 8B, respectively, of the main text. The readout parameter for image quantification was the signal, which represents "*the sum of the individual pixel intensity values for a shape minus the product of the average intensity values of the pixels in the background and the total number of pixels enclosed by the shape*" (Calculation Descriptions within the help of the Image Studio v.5.2.5 software). The magenta rectangle displays the area used as the background. The total signal of each lane is represented by the red rectangle covering the whole area of the lane. Shapes of "oligomers", "slow-/intermediate-migrating," and "fast-migrating" species were manually added and adjusted in size and position. **C**) SDS-PAGE was done on 7% separating gel. Both the gel and the running buffer contained neither GTP nor $MgCl_2$. Samples of 8.23 µM self-produced hTGase 2 were first incubated for 30 min at 20 °C in 100 mM MOPS pH 8.0, 100 mM NaCl, 2.12% (v/v) DMSO, 5.33% (v/v) glycerol with $CaCl_2$, GTP, $MgCl_2$, and different concentrations of inhibitor **2** as indicated. GTP and $MgCl_2$ were then added as indicated, and all samples were again incubated for 60 min at 20 °C. After addition of SDS loading buffer and denaturation for 5 min at 60 °C, a volume of 3 µL of each sample containing 1.5 µg of hTGase 2 was loaded onto the gel. After the electrophoretic separation, gels were stained with Coomassie Brilliant Blue G250 and scanned. M, 3 µL of PageRuler™ Plus Prestained Protein Ladder (neither treated with SDS loading buffer nor denaturated); SF, solvent front.

A	native GTP-PAGE													
lane	1	2	3	4	5	6	7	8	9	10	11	12	13	14
3 mM Ca^{2+}			x	x	x	x	x	x	x	x	x	x	x	x
500 µM GTP		x	x	x										
1 mM Mg^{2+}			x											
inhibitor **2** (µM)	0	0	0	0	0	0.425	0.85	1.7	2.55	4.25	5.74	8.5	25.5	85
30 min incubation at 20 °C														
500 µM GTP	x				x	x	x	x	x	x	x	x	x	x
1 mM Mg^{2+}														
60 min incubation at 20 °C then addition of loading buffer														

B	native GTP-PAGE													
lane	1	2	3	4	5	6	7	8	9	10	11	12	13	14
3 mM Ca^{2+}			x	x	x	x	x	x	x	x	x	x	x	x
500 µM GTP		x	x	x										
1 mM Mg^{2+}		x	x											
inhibitor **2** (µM)	0	0	0	0	0	0.425	0.85	1.7	2.55	4.25	5.74	8.5	25.5	85
30 min incubation at 20 °C														
500 µM GTP	x				x	x	x	x	x	x	x	x	x	x
1 mM Mg^{2+}	x				x	x	x	x	x	x	x	x	x	x
60 min incubation at 20 °C then addition of loading buffer														

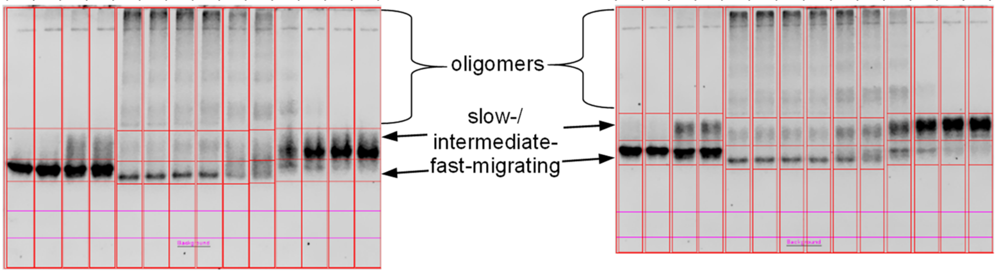

C	SDS-PAGE														
lane	1	2	3	4	5	6	7	8	9	10	11	12	13	14	M
3 mM Ca^{2+}			x	x	x	x	x	x	x	x	x	x	x	x	
500 µM GTP		x	x	x											
1 mM Mg^{2+}		x	x												
inhibitor **2** (µM)	0	0	0	0	0	0.425	0.85	1.7	2.55	4.25	5.74	8.5	25.5	85	
30 min incubation at 20 °C															
500 µM GTP	x				x	x	x	x	x	x	x	x	x	x	
1 mM Mg^{2+}	x				x	x	x	x	x	x	x	x	x	x	
60 min incubation at 20 °C, then addition of SDS loading buffer and sample denaturation for 5 min at 60 °C															

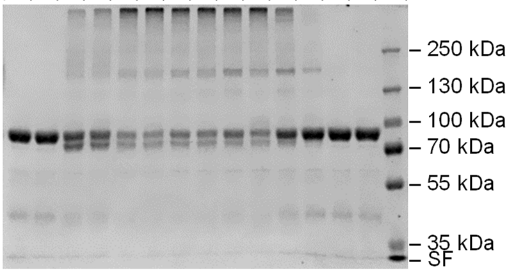

Figure S8. Calculation of the net charge of in-house produced *N*-terminally Twin-Strep-tagged hTGase 2 at a pH of 8.3.

The depicted single-letter amino acid sequence of in-house produced *N*-terminally Twin-Strep-tagged hTGase 2 was based on the results from DNA sequencing (Eurofins Genomics Germany GmbH) of the respective plasmid pPSG-IBA105/TCS-hTGase 2. The single-letter amino acid sequence was analyzed by the web-based bioinformatics tool Prot pi (release: 2.2.29.150, https://www.protpi.ch/) to obtain a net charge of -37.8 at a pH of 8.3.

```
        10         20         30         40         50         60         70
MASAWSHPQF EKGGGSGGGS GGSAWSHPQF EKSGMAEELV LERCDLELET NGRDHHTADL CREKLVVRRG

        80         90        100        110        120        130        140
QPFWLTLHFE GRNYEASVDS LTFSVVTGPA PSQEAGTKAR FPLRDAVEEG DWTATVVDQQ DCTLSLQLTT

       150        160        170        180        190        200        210
PANAPIGLYR LSLEASTGYQ GSSFVLGHFI LLFNAWCPAD AVYLDSEEER QEYVLTQQGF IYQGSAKFIK

       220        230        240        250        260        270        280
NIPWNFGQFE DGILDICLIL LDVNPKFLKN AGRDCSRRSS PVYVGRVVSG MVNCNDDQGV LLGRWDNNYG

       290        300        310        320        330        340        350
DGVSPMSWIG SVDILRRWKN HGCQRVKYGQ CWVFAAVACT VLRCLGIPTR VVTNYNSAHD QNSNLLIEYF

       360        370        380        390        400        410        420
RNEFGEIQGD KSEMIWNFHC WVESWMTRPD LQPGYEGWQA LDPTPQEKSE GTYCCGPVPV RAIKEGDLST

       430        440        450        460        470        480        490
KYDAPFVFAE VNADVVDWIQ QDDGSVHKSI NRSLIVGLKI STKSVGRDER EDITHTYKYP EGSSEEREAF

       500        510        520        530        540        550        560
TRANHLNKLA EKEETGMAMR IRVGQSMNMG SDFDVFAHIT NNTAEEYVCR LLLCARTVSY NGILGPECGT

       570        580        590        600        610        620        630
KYLLNLNLEP FSEKSVPLCI LYEKYRDCLT ESNLIKVRAL LVEPVINSYL LAERDLYLEN PEIKIRILGE

       640        650        660        670        680        690        700
PKQKRKLVAE VSLQNPLPVA LEGCTFTVEG AGLTEEQKTV EIPDPVEAGE EVKVRMDLLP LHMGLHKLVV

       710        720 721
NFESDKLKAV KGFRNVIIGP   A
```

References

1. Conte, S. D. and de Boor, C., *Elementary Numerical Analysis*, Philadelphia (PA), 2017.
2. Otto, M., *Chemometrics: Statistics and Computer Application in Analytical Chemistry*, Wiley-VCH, Weinheim (Germany), 2016.
3. Kim, N.; Lee, W. K.; Lee, S. H.; Jin, K. S.; Kim, K. H.; Lee, Y.; Song, M. and Kim, S. Y. Inter-molecular crosslinking activity is engendered by the dimeric form of transglutaminase 2. *Amino Acids*, **2017**, *49*, 461-471.
4. Hauser, C.; Wodtke, R.; Löser, R. and Pietsch, M. A fluorescence anisotropy-based assay for determining the activity of tissue transglutaminase. *Amino Acids*, **2017**, *49*, 567-583.
